# Hearing Loss Controlled by Optogenetic Stimulation of Nonexcitable Nonglial Cells in the Cochlea of the Inner Ear

**DOI:** 10.3389/fnmol.2017.00300

**Published:** 2017-09-21

**Authors:** Mitsuo P. Sato, Taiga Higuchi, Fumiaki Nin, Genki Ogata, Seishiro Sawamura, Takamasa Yoshida, Takeru Ota, Karin Hori, Shizuo Komune, Satoru Uetsuka, Samuel Choi, Masatsugu Masuda, Takahisa Watabe, Sho Kanzaki, Kaoru Ogawa, Hidenori Inohara, Shuichi Sakamoto, Hirohide Takebayashi, Katsumi Doi, Kenji F. Tanaka, Hiroshi Hibino

**Affiliations:** ^1^Department of Molecular Physiology, Niigata University School of Medicine Niigata, Japan; ^2^Department of Otolaryngology, Kindai University Faculty of Medicine Osaka, Japan; ^3^Center for Transdisciplinary Research, Niigata University Niigata, Japan; ^4^Department of Otorhinolaryngology, Graduate School of Medical Sciences, Kyushu University Fukuoka, Japan; ^5^Division of Otolaryngology—Head and Neck Surgery, Yuaikai Oda Hospital Saga, Japan; ^6^Department of Otorhinolaryngology—Head and Neck Surgery, Graduate School of Medicine, Osaka University Osaka, Japan; ^7^Department of Electrical and Electronics Engineering, Niigata University Niigata, Japan; ^8^AMED-CREST, AMED Niigata, Japan; ^9^Department of Otolaryngology, Kyorin University School of Medicine Tokyo, Japan; ^10^Department of Otolaryngology, Head and Neck Surgery, Keio University School of Medicine Tokyo, Japan; ^11^Department of Mechanical and Production Engineering, Niigata University Niigata, Japan; ^12^Division of Neurobiology and Anatomy, Graduate School of Medical and Dental Sciences, Niigata University Niigata, Japan; ^13^Department of Neuropsychiatry, Keio University School of Medicine Tokyo, Japan

**Keywords:** cochlea, sensorineural hearing loss, endocochlear potential, nonexcitable cell, optogenetics

## Abstract

Light-gated ion channels and transporters have been applied to a broad array of excitable cells including neurons, cardiac myocytes, skeletal muscle cells and pancreatic β-cells in an organism to clarify their physiological and pathological roles. Nonetheless, among nonexcitable cells, only glial cells have been studied *in vivo* by this approach. Here, by optogenetic stimulation of a different nonexcitable cell type in the cochlea of the inner ear, we induce and control hearing loss. To our knowledge, deafness animal models using optogenetics have not yet been established. Analysis of transgenic mice expressing channelrhodopsin-2 (ChR2) induced by an oligodendrocyte-specific promoter identified this channel in nonglial cells—melanocytes—of an epithelial-like tissue in the cochlea. The membrane potential of these cells underlies a highly positive potential in a K^+^-rich extracellular solution, endolymph; this electrical property is essential for hearing. Illumination of the cochlea to activate ChR2 and depolarize the melanocytes significantly impaired hearing within a few minutes, accompanied by a reduction in the endolymphatic potential. After cessation of the illumination, the hearing thresholds and potential returned to baseline during several minutes. These responses were replicable multiple times. ChR2 was also expressed in cochlear glial cells surrounding the neuronal components, but slight neural activation caused by the optical stimulation was unlikely to be involved in the hearing impairment. The acute-onset, reversible and repeatable phenotype, which is inaccessible to conventional gene-targeting and pharmacological approaches, seems to at least partially resemble the symptom in a population of patients with sensorineural hearing loss. Taken together, this mouse line may not only broaden applications of optogenetics but also contribute to the progress of translational research on deafness.

## Introduction

In biomedical research, opsins, which are light-gated ion channels or transporters such as cation-selective channelrhodopsin and archaerhodopsin and anion-permeable halorhodopsin, have been expressed primarily in neuronal cell types* in vivo* (Deisseroth, 2015; Glock et al., 2015). Temporal and spatial control of the opsin activity with light has unveiled diverse functional roles of different neurons as well as key cellular mechanisms underlying construction of neural circuits and networks in the brain (Grosenick et al., 2015). This optogenetic approach has also highlighted several pathophysiological phenotypes in nervous systems and showed their possible causes. Moreover, technical advances have allowed researchers to induce light-gated channels in cardiac myocytes, skeletal muscle cells and pancreatic β-cells in live animals and to electrically manipulate the cells in a particular region and/or timing with illumination (Bruegmann et al., 2015; Vogt et al., 2015; Johnston et al., 2016). These experiments have provided insights into novel therapies for heart diseases, muscle paralysis and diabetes. Besides these excitable cells, glial cells, which are nonexcitable, have recently been analyzed with optogenetics *in vivo*. Tanaka et al. (2012) developed a transgenic methodology that can stably and strongly induce the channelrhodopsin-2 (ChR2) protein in the mouse; this approach is called “the knockin-mediated enhanced gene expression by improved tetracycline-controlled gene induction (KENGE-tet) system”. In this system, a promoter that is active in a different cell population is selectable; indeed, the *ChR2* gene is reported to be driven in glial cells of astrocytes, oligodendrocytes, or microglia. In a mouse line harboring ChR2 in oligodendrocytes, photodepolarization of these cells causes early- and late-onset acceleration of axonal conduction and affects short- and long-term functional plasticity in the hippocampus (Yamazaki et al., 2014).

In spite of these achievements, nonexcitable cell types other than glial cells have not yet been studied in an organism with opsins. The proteolipid protein (PLP) promoter, which is used to induce ChR2 in oligodendrocytes in the KENGE-tet system (Tanaka et al., 2012), has a transcriptional activity in an epithelial-like tissue, the stria vascularis (StV), of the mammalian cochlea (Morris et al., 2006; Inamura et al., 2012). The StV plays central roles in formation of a highly positive potential in the K^+^-rich extracellular solution, endolymph (Zdebik et al., 2009); this potential underlies marked sensitivity of sensory hair cells and thus is essential for hearing (Honrubia and Ward, 1969; Jacob et al., 2011). To expand applications and significance of optogenetics, in the present study we focused our analyses on the cochlea of a mouse line expressing ChR2 under control of the PLP promoter. Unexpectedly, ChR2 was detected in nonglial cells, melanocytes, in the StV. Hearing phenotypes that result from optical control of ChR2 have not been replicated in animals by any conventional gene-targeting or pharmacological approaches. Stimulation of the cochlea with blue light to depolarize the melanocytes caused significant hearing loss within a few minutes. The deafness stemmed primarily from a reduction in the endolymphatic potential. The potential and hearing completely recovered soon after the cessation of illumination. These responses were repeatable. Because the patterns of deafness observed in the ChR2-expressing mouse at least partially mimicked those in idiopathic sensorineural hearing loss in humans, this animal model may not only increase the repertoire of optogenetic targets but also serve as a platform for elucidation of the pathological processes of various inner ear diseases and for development of medical treatments.

## Materials and Methods

### Ethical Statement for Animal Experiments

All the experimental protocols involving animals were approved by the Animal Research Committees of Niigata University School of Medicine. Experiments were conducted under the supervision of the Committees and in accordance with the Guidelines for Animal Experiments of Niigata University and the Japanese Animal Protection and Management Law. All animal handling and reporting comply with the ARRIVE guidelines (Kilkenny et al., 2010).

### Transgenic Animals and General Information for Experiments

The procedure for generation of the transgenic mice was described previously (Tanaka et al., 2012). Briefly, two mouse lines were prepared: PLP-tTA (transcriptional activator, tTA) mice, in which expression of a tetracycline-controlled tTA protein is driven by the PLP promoter, and tetO-ChR2(C128S)-enhanced yellow fluorescence protein (EYFP) knock-in (tetO) mice, in which genes encoding ChR2(C128S) and EYFP are integrated into the genome. These mice were crossed to generate bigenic mice stably expressing EYFP-fused ChR2(C128S). The mice were housed at the animal facility of the Niigata University and kept in a 12-h light/12-h dark cycle. Food and water were provided *ad libitum*. Experiments were conducted during the light period of the cycle. The smallest number of animals possible was used in this study, and every effort was made to minimize their suffering. Animals were randomly assigned to each experiment. No blinding was performed.

In the present study, wild-type (WT), tTA, tetO and bigenic mice of either sex at postnatal 6–10 weeks of age (weeks P6–P10) were used; they were initially anesthetized by intraperitoneal injection of urethane (1.5 g/kg). The toe pinch, corneal reflexes and respiratory rate were used as guidelines to evaluate the depth of anesthesia. When anesthesia was not sufficient, urethane (0.3 g/kg) was additionally provided to the animals. For measurements of the auditory brainstem response (ABR) and potential in endolymph, after deep anesthesia was confirmed, each mouse underwent a tracheotomy and was subjected to one experimental procedure (see below). The mice were maintained at approximately 37°C using a heating blanket (BWT-100A, Bio Research Center, Nagoya, Japan) with spontaneous respiration throughout the experiments. The animals were finally euthanized with an overdose of sodium pentobarbital (200 mg/kg) by intraperitoneal injection.

### Western Blotting

Several tissue types were dissected and excised from the deeply anesthetized animals (week P10) as follows: two cochleae, the whole brain, the liver, and a kidney from a single bigenic mouse, two cochleae and the whole brain from a single WT mouse, and two cochleae from a single tTA or tetO mouse. These tissues were flash-frozen in liquid nitrogen and kept at −80 °C until western blot analysis. The frozen cochleae and other tissues were homogenized in 0.5 and 2 ml of RIPA buffer (Wako, Osaka, Japan) supplemented with cOmplete™ Protease Inhibitor Cocktail (1 tablet per 50 ml; Roche, Basel, Switzerland), respectively. Protein concentrations in the tissue lysates were determined by means of the BCA Protein Assay Kit (Thermo Fisher Scientific, MA, USA). Prior to electrophoresis, 4.5 μg of each lysate was denatured in Laemmli Sample Buffer (Bio-Rad Laboratories, CA, USA) containing 50 mM dithiothreitol (Wako). Thereafter, the samples were resolved on a 10% Mini-PROTEAN TGX gel (Bio-Rad Laboratories) and transferred to a polyvinylidene difluoride membrane (Hybond-P membrane; GE Healthcare, Buckinghamshire, England) using a Mini-PROTEAN Tetra Cell System (Bio-Rad Laboratories). To confirm successful transfer of the proteins, the membrane was incubated for 5 min in a solution containing 0.1% of Ponceau S (Sigma, MO, USA) and 5% of acetic acid (Wako), and then washed with ultrapure water for 2 min. The image was acquired by a digital camera (PowerShot G11; Canon, Tokyo, Japan). After destaining with TBS-T (20 mM Tris-HCl pH 7.4, 150 mM NaCl, 0.1% of Tween 20, pH 7.5) for 30 min, the membrane was treated with Blocking One buffer (Nacalai Tesque, Kyoto, Japan) for 1 h at room temperature and subsequently incubated overnight at 4°C with a monoclonal antibody against green fluorescent protein (GFP; 1:1000; G10362; Thermo Fisher Scientific). Next, the membrane was reacted with a peroxidase-conjugated goat anti-rabbit antibody (1:2000; #32460; Thermo Fisher Scientific). Finally, the immunoreactivity was visualized by chemiluminescence using the ECL prime substrate (GE Healthcare) and recorded as images with a ChemiDoc XRS system (Bio-Rad Laboratories).

### Histochemistry

Tissue section immunohistochemical analysis of cochleae of bigenic mice was performed as described previously (Hibino et al., 1997). The anesthetized mice were infused with phosphate-buffered saline (PBS) through the left ventricle and subsequently with 4% paraformaldehyde (PFA) dissolved in PBS. The composition of PBS was 137.0 mM NaCl, 2.7 mM KCl, 8 mM Na_2_HPO_4_·12H_2_O, 1.4 mM KH_2_PO_4_, pH 7.4. The cochleae were excised from the temporal bone and treated with 4% PFA overnight at 4°C. The samples were then rinsed with PBS and incubated in a 30% sucrose solution overnight at 4°C with gentle stirring. After that, they were immersed in the SCEM (Leica, Microsystem, Wetzlar, Germany) and rapidly frozen. Cryosections (5-μm thickness) of the cochleae were made by Kawamoto’s film method (Kawamoto and Shimizu, 2000). For visualizing the YFP protein with diamino benzidine (DAB), the tissue slices were pretreated with 1% hydrogen peroxide in 99% ethanol for 30 min to inactivate endogenous peroxidases and incubated with the blocking reagent of the ABC kit (Vector Laboratory, CA, USA) and then with an antibody against GFP (1:1000; Frontier Institute, Hokkaido, Japan) overnight at 4°C. They were incubated for 30 min at room temperature with a biotinylated secondary antibody solution from the ABC kit, followed by several washes with PBS. The samples were finally embedded with a mounting medium (SCMM; Leica, Microsystem, Wetzlar, Germany). For immunolabeling assays, the slices, which had been pretreated with a blocking solution (Blocking One) for 1 h at room temperature, were incubated overnight at 4°C with antibodies against GLUT1 (1:100; # sc-1605; Santa Cruz, CA, USA), Kir4.1 (1:800; Alomone, Jerusalem, Israel), barttin (1:200; kindly provided by Dr. Uchida), neurofilament M (1:200; #AB1987; Merck Millipore, Darmstadt, Germany), or myelin basic protein (1:50; #AB980; Merck Millipore, Darmstadt, Germany) and then incubated for 1 h at room temperature with a tetramethylrhodamine isothiocyanate (TRITC)-conjugated anti-goat IgG (for GLUT1) or anti-rabbit IgG secondary antibodies (for Kir4.1, barttin, neurofilament M and myelin basic protein; Jackson ImmunoResearch Laboratories, Inc., PA, USA). The samples were finally immersed in a mounting medium containing DAPI (VECTASHIELD; Vector Laboratory, CA, USA).

Cochlear cross-sections of WT mice were also prepared as described above, and these samples as well as the cross-sections of bigenic mice were stained with hematoxylin and eosin (HE; Muto Pure Chemicals Co., Ltd., Tokyo, Japan). The specimens were then examined to assess the morphology of the cochleae. In this context, measurement of the thickness of the StV and calculation of the number of cell bodies of spiral ganglions (SGs) were performed by evaluating images captured by means of a light microscope. Of note, in each slice “thickness” was determined as an average of three distance measurements perpendicular to the surface of marginal cells (MC) facing the scala media (SM).

Immunolabeling assays with the isolated cells were carried out on the basis of an earlier report (Ando et al., 2008). Cells dissociated from the StV (for detailed Methods, see below), were placed on cover glasses, and fixed with 4% PFA in PBS overnight at 4°C. Thereafter, the samples were treated with the blocking solution, incubated with antibodies against Kir4.1 and barttin, followed by TRITC-conjugated anti-rabbit secondary antibodies and the mounting medium containing DAPI by the same procedure used for the aforementioned immunolabeling assays.

Immunolabeling assays of the organ of Corti (OC) were conducted as described previously (Park et al., 2010). In brief, cochleae excised from bigenic and WT mice were fixed with 4% PFA in PBS overnight at 4°C. After decalcification of the cochleae with 120 mM EDTA at pH 7.4 for 3 days, tissue composed of the sensory epithelia including the OC and the spiral limbus was dissociated carefully from each organ and then blocked with 5% BSA and 0.3% Triton X-100 in PBS. The specimens were incubated overnight at 4°C with rhodamine phalloidin (1:250; R415; Thermo Fisher Scientific, MA, USA) and antibodies against myosin VIIa (1:300; Proteus Biosciences, CA, USA). After a wash with PBS, the specimens were incubated with anti-rabbit IgG secondary antibodies (conjugated with Alexa 488) for 1 h at room temperature and then mounted in a medium containing DAPI on a slide glass.

Finally, images of the stained and labeled samples were captured by means of a bright-field light microscope (FSX100, Olympus, A-01, Tokyo, Japan) or a laser-scanning confocal microscope (Nikon ECLIPSE Ti, Nikon, A-01, Tokyo, Japan).

It should be noted that all the histochemical experiments described above were designed to confirm the expression of ChR2(C128S) and to determine its distribution and localization in the cochleae of bigenic mice. Therefore, before the experiments, basic effects of optical stimulation on the auditory thresholds and endolymphatic potential (see below) were not examined. As shown in Table 1, multiple animals were used for the histochemical assays; from these results, only representative images are displayed in the main and Supplementary Figures.

**Table 1:** Numbers of mice used for histochemical experiments.

Mouse genotypes	Display items	Number of mice used	Total number of mice
Bigenic	Figure 2A	4	41
	Figures 2B,C	2	
	Supplementary Figures S2B,C	5	
	Supplementary Figures S3E–H	3	
	Supplementary Figure S4	7	
	Supplementary Figure S5	8	
	Supplementary Figure S6	8*	
	Supplementary Figure S10	2^#^	
	Supplementary Figure S13	2^#^	
Wild type	Supplementary Figures S2A,C	5	8
	Supplementary Figures S3A–D	3	

For each series of experiments, only representative image data are displayed in the main and Supplementary Figures (Figures 2A–C and Supplementary Figures S2A, S2B, S3A–H, S4A–C, S5A–C, S6A, S10, and S13). *Five mice were at week P6, and three mice were at week P10. ^#^One was subjected to the auditory brainstem response (ABR) measurement and the other was used for the endocochlear potential (EP) recording, in advance (see Tables 2, 3 and “Materials and Methods” Section *themaintext*).

### Isolation of Cells from the Stria Vascularis

All the following procedures were performed in a dark room under a stereoscope. Cochleae dissected from the temporal bone were vertically split into a few pieces containing the lateral cochlear bone in chilled Tyrode’s solution consisting of 140 mM NaCl, 3.6 mM KCl, 1 mM CaCl_2_, 1.0 mM MgCl_2_, 2.6 mM Tris, 6 mM HEPES, 10 mM D-glucose, and 10 mM Na-pyruvate. Next, tissue strips of the StV were peeled off from the cochlear bone with a fine needle under a stereomicroscope, rinsed with Tyrode’s solution without Ca^2+^ and Mg^2+^, and then incubated in a 0.25% Trypsin-EDTA solution (Wako) for 10 min at 37°C. Subsequently, the tissues were treated with Tyrode’s solution containing 0.5 mg/ml DNase I (Sigma), 1 mg/ml BSA (Sigma) and cOmplete™ Protease Inhibitor Cocktail, for 10 min at 4°C. Enzymatic reactions were terminated by washing the tissues with chilled Tyrode’s solution. To isolate strial cells, the samples were pipetted gently (10 times). The solution containing the cells was placed on a cover glass coated with Cell-Tak (BD Biosciences, Franklin Lakes, NJ, USA) and used for immunolabeling experiments and patch-clamp assays.

### Auditory Brainstem Response (ABR) Measurements

ABR was measured basically as previously described (Wu et al., 2004). The mice, after being deeply anesthetized by intraperitoneal injection of urethane (1.5 g/kg) and undergoing tracheotomy, were placed in a prone or supine position in a box that was acoustically as well as electrically shielded. Stainless-steel needle electrodes were subcutaneously inserted in the parietal region (noninverting), under the pinnae (inverting), and in the posterior region of the neck (reference). For acoustic stimuli, either 100-μs clicks or 2-ms tone bursts of 4.0, 8.5, 12.5, 16.8, 25.0, or 33.3 kHz containing a 0.5-ms rising phase and 0.5-ms falling phases were generated by means of a speaker (STAX SRM-323S; SR-307, Saitama, Japan). After a microphone (ACO, TYPE4116, Tokyo, Japan) was calibrated with a sound calibrator (ACO, TYPE2127, Tokyo, Japan) inside the box, it was placed beside the left ear of a mouse. Because the speaker was equidistant from the ear and the microphone, we could determine the sound pressure level (SPL) that was perceived by the mice in this system. Click intensities are expressed as a peak equivalent SPL, which is defined as the SPL of a 3-kHz reference tone adjusted so that its peak amplitude matches that of the click (Coats et al., 1979). Individual signals emitted from the auditory tract were amplified 5000-fold and processed with a band-pass filter (0.3–2 kHz) in an amplifier (EX-1 Differential Amplifiers; Dagon Corporation, MN, USA). Then, these data were digitized, processed again with a band-pass filter (0.3–2 kHz), and averaged (1000 sweeps; LabVIEW 2013 SP1; National Instruments, TX, USA). The stimulus sounds at 50 dB SPL (4.0 kHz and click), 30 dB SPL (8.5, 12.5, 16.8, or 25.0 kHz), or 40 dB SPL (33.3 kHz) were initially provided for the mice and thereafter were decreased in 10-dB or 5-dB steps until a hearing threshold was determined. Because the noise level in our ABR settings was 0–10 dB, the minimum SPL we could record was 10 dB.

Some animals underwent the surgical procedure that exposed their bulla for illumination; the capsule of the bulla was not opened. Then, they were placed in a supine position for ABR measurements. For optical stimulation of each cochlea, blue light (peak wavelength 463 nm) was provided through a thin wall of the bulla from a connectorized single LED (93 mW; Doric Lenses, Inc., Ville de Québec, QC, Canada). Therefore, the cochlea inside the bulla was intact throughout the ABR measurements. Light intensity was controlled by changing the distance between the LED probe and the surface of the bulla (distance; 1.0–30 mm) and by providing irradiance of 0.1–1.0 mW/mm^2^.

Both 129S6/SvEvTac and C57BL/6J mouse strains, which were used for generating the transgenic mouse lines, have a genetic background of high-frequency hearing loss. Hearing thresholds of C57BL/6J mice begin to increase from P5 months (Fujita et al., 2015). In 129S6/SvEvTac mouse lines, hearing loss is inherited in an autosomal recessive manner, although in some cases, the auditory-threshold elevation can be moderately detectable even at week P4, after which it progresses minimally during the subsequent several weeks (Peguero and Tempel, 2015). Accordingly, in this study, we analyzed mice at weeks P6–P10. Prior to experiments with optical stimulation, we measured ABR thresholds of all the mice with tone-burst sound stimuli at 4.0, 8.5, 12.5, 16.8, 25.0 and 33.3 kHz. Of 34 bigenic mice tested, six showed moderate hearing loss only at high frequencies (33.3 kHz). The mice with normal hearing (*n* = 28) were subjected to other experiments as shown in Table 2. A similar hearing loss was also observed in three out of 21 littermate WT mice, three out of 13 tTA mice, and two out of 12 tetO mice. Only mice with normal hearing were used for further assays (Table 2).

**Table 2:** Numbers of mice used for ABR measurements with optical stimulation.

Mouse genotypes	Display items	Number of mice used	Total number of mice
Bigenic	Figure 3 and	6	28
	Supplementary Figure S8D		
	Supplementary Figure S8A	4	
	Supplementary Figure S8B	4	
	Supplementary Figure S8C	10*	
	Supplementary Figure S9A	6	
	Supplementary Figures S10A,B	1	
	Supplementary Figure S10C	1	
Wild-type	Supplementary Figure S7	5^#^	18
	Supplementary Figure S8C	8	
	Supplementary Figure S9B	5	
tTA	Supplementary Figure S8C	5	10
	Supplementary Figure S9C	5	
tetO	Supplementary Figure S8C	5	10
	Supplementary Figure S9D	5	

ABR thresholds of all the mice under control conditions (without illumination) were normal throughout tested six different frequencies of tone-burst sound stimuli. *Four mice assayed with illumination at 0.45 mV/mm^2^ were same as the animals used in Supplementary Figure S8B. ^#^Optical stimuli were not provided (see legend of Supplementary Figure S7).

In some bigenic mice, restoration of hearing after optical stimulation was examined as follows. After an interval of 5 min, the mice were given click or tone burst stimuli at the same intensity as the auditory thresholds obtained under control conditions. When the brainstem responses were detected, the animals were exposed to the sounds at decreasing intensities to confirm their auditory thresholds. Alternatively, when no ABR signal was detected, the stimuli were increased by 5 dB SPL. Audition of all 12 individuals tested was successfully evaluated with either protocol, which took 2 min each at most.

### Electrical ABR Measurements

To measure electrical ABR (eABR), the procedure described in another study (Yamane et al., 1981) was modified and used. Five WT mice and five bigenic mice were tested. Prior to the eABR measurements, the endolymphatic potential was assayed (for procedures, see below). First, three stainless-steel needle electrodes were subcutaneously inserted as described in aforementioned “*ABR Measurements*” Section. Next, in each mouse, the bulla was surgically accessed by a ventrolateral approach. Furthermore, the capsule of the bulla was partially removed and the cochlea was exposed. Two fenestrae were made at the basal turn and apex of the bony wall of the cochlea. After that, approximately 30 μl of a bumetanide solution (250 μM), which was prepared by diluting a 100 mM stock solution (in 0.1 M NaOH) 400-fold with saline, was applied to the interior of the bulla (Higashiyama et al., 2003). A glass microelectrode was advanced through the fenestra at the basal turn and placed in the endolymph of the SM to record the endocochlear potential (EP; for a detailed procedure, see *“Measurement of the Endolymphatic Potential”* Section described below). After the EP reached values less than −5 mV, which indicate sufficient dysfunction of the StV, we electrically stimulated the cochlea. Two electrodes containing an Ag/AgCl wire covered with Sylgard^®^ 184 Silicone Elastomer (Dow Corning Corporation, MI, USA) except for the tip were inserted into perilymph (PL) through the fenestra at the apex and the round window membrane, respectively. Electrical stimuli of biphasic rectangular pulses (50-μs duration, frequency 10 Hz) generated by an electronic stimulator (SEN-3301; Nihon Kohden SEN-3301, Tokyo, Japan) and an isolator (SS-403J; Nihon Kohden) were administered to the cochlea through the Ag/AgCl wire electrodes.

Although the responses from the brain were recorded with the amplifier and the analytical software used in ABR assays, some of the conditions for data processing were modified as follows: the recorded individual responses were amplified 10,000-fold, processed with a band-pass filter of 10 Hz to 10 kHz, and subjected to averaging of 200 sweeps. The current to electrically stimulate the cochlea started from 750 μA to 800 μA and was decreased in 50-μA steps until a threshold was determined. In each eABR trace, the III wave was identified with reference to an earlier work (Yamane et al., 1981).

For optical activation of ChR2(C128S), blue light (463 nm, 0.45 mW/mm^2^) was applied to the exposed cochlea with the same tool and procedure as in the aforementioned ABR measurements. Most of the light should stimulate the interior of the cochlea across its bony wall; however, a small fraction of the light was likely to reach the inside through the multiple fenestrae.

### Patch-Clamp Recordings

Mice of different transgenic lines were subjected to isolation of the strial cells for their electrophysiological characterization. In general, a photoinduced response of ChR2(C128S) decays as its optical stimulation is repeated (Schoenenberger et al., 2009). To record the response by the patch-clamp technique as much as possible, effects of illumination of the cochlea on ABR thresholds were not examined. Whole-cell patch-clamp analyses were performed as reported previously (Takeuchi and Ando, 1998; Higuchi et al., 2014). Glass electrodes, whose tip resistance was 1–3 MΩ, were filled with a pipette solution consisting of 135 mM KCl, 5 mM EGTA, 3.1 mM MgCl_2_, 6 mM HEPES, 2 mM Na_2_-ATP, and 0.1 mM spermine (pH 7.3). Cells dissociated from the StV were bathed in a solution composed of 114 mM NaCl, 3.6 mM KCl, 26 mM NaHCO_3_, 1.25 mM NaH_2_PO_4_, 1 mM CaCl_2_, 1 mM MgCl_2_, 10 mM D-glucose and 10 mM sodium pyruvate. The pH level of this bathing solution was adjusted to 7.3 with 95% O_2_/5% CO_2_ gas. Types of dissociated cells were determined by their characteristic morphological features under a microscope. Liquid junction potentials between the pipette and bathing solutions were adjusted to zero, and after that, the electrode was attached to a cell. The light of the microscope was turned off as soon as the seal resistance reached ~1 GΩ, and next, the whole-cell configuration was set up. Membrane potentials and currents were measured at a holding current of 0 pA and a holding potential of −80 mV using a patch-clamp amplifier (Axopatch 200B; Molecular Devices, CA, USA) and commercially available software (pClamp10.5; Molecular Devices). During the recording, blue light (peak wavelength 463 nm) transiently illuminated the cells to activate ChR2 for 30 s from a light source (Doric Lenses, Inc., Ville de Québec, QC, Canada) that was located 15 mm away from the chamber. Under these conditions, irradiance of the light shining on the surface of the strial cells was 0.45 mW/mm^2^. In every patch-clamp assay, the blue light was switched to green light (peak wavelength 535 nm, 100 W; Nikon Diaphoto 300, Tokyo, Japan) to rapidly close ChR2. The acquired data on membrane potentials and currents were reproduced with a low-pass filter of 1 kHz and digitized at a sampling rate of 10 kHz using DigiData1440 (Molecular Devices). The numbers of the animals used for the patch-clamp experiments described above were as follows: nine WT mice, eight tTA mice, eight tetO mice and nine bigenic mice. Note that in some of these mice, isolation of the strial cells or electrophysiological recording was not successful.

### Measurement of the Endolymphatic Potential

The endolymphatic potential was measured in accordance with the procedures used in our earlier works (Hibino et al., 1997; Nin et al., 2008). Microelectrodes, which were fabricated from single-barreled glass capillaries (WPI, Sarasota, FL, USA), were filled with 150 mM KCl and connected via an Ag/AgCl electrode to an electrometer (FD223a; WPI). The cochleae in WT and transgenic mice were exposed by a ventrolateral approach. A fenestra of roughly 100 μm in diameter was made on the bony wall of the basal cochlear turn using a micro chisel. A microelectrode was advanced from PL through the fenestra using a micromanipulator (MP-285; Sutter Instrument Co., Novato, CA, USA) and was held in the endolymph of the SM to continuously monitor the EP. An Ag/AgCl wire was inserted into the abdominal muscles and used as a reference. Potentials were measured with respect to PL, which was defined as 0 mV. After the potential values were stabilized, the cochleae were optically stimulated. Light (463 nm) was shone on the surface of the cochlear bony wall with the aforementioned LED. Intensity of the illumination was controlled by changing the distance between the LED probe and bony wall (7.5–60 mm), providing irradiance of 0.02–0.75 mW/mm^2^. Most of the light should illuminate the interior of the cochlea across its bony wall; however, a small fraction of the light was likely to penetrate through the fenestra for insertion of the microelectrode. Finally, the microelectrode was pulled back to PL, and drifts in potential measurements were confirmed to be less than 3 mV. As shown in Table 3, multiple animals were used for the measurements.

**Table 3:** Numbers of mice used for recordings of the endocochlear potential.

Mouse genotypes	Display items	Number of mice used	Total number of mice
Bigenic	Figures 5A,B	6	26
	Supplementary Figure S12A	12*	
	Supplementary Figure S12B	5^#^	
	Supplementary Figure S12C	1	
	Supplementary Figures S13A,B	1	
	Supplementary Figure S13C	1	
Wild-type	Figures 5A,B	5	5
tTA	Figures 5A,B	5	5
tetO	Figures 5A,B	5	5

*For each of the four series of the assays (illumination: 0.02, 0.1, 0.45 and 0.75 mV/mm^2^), three individual animals were used. ^#^A representative trace of the EP recording is shown in the left panel of Supplementary Figure S12B.

### Statistical Analysis

Statistical significance was assessed with unpaired or paired two-tailed Student’s *t* test to compare two conditions or an analysis of variance (ANOVA) for comparison among multiple experimental conditions followed by the Bonferroni correction or Tukey’s *post hoc* test for comparison between conditions. To compare data with a control, Dunnett’s test was used. Statistical tests were carried out and plots were built in the GraphPad Prism 6 software. All *n* and *P* values are indicated in the main text and figures. No statistical analyses were conducted to predetermine sample sizes, but our sample sizes are similar to those generally employed in the field.

## Results

### Expression of ChR2(C128S) in the Cochlea

**Figure 1:**
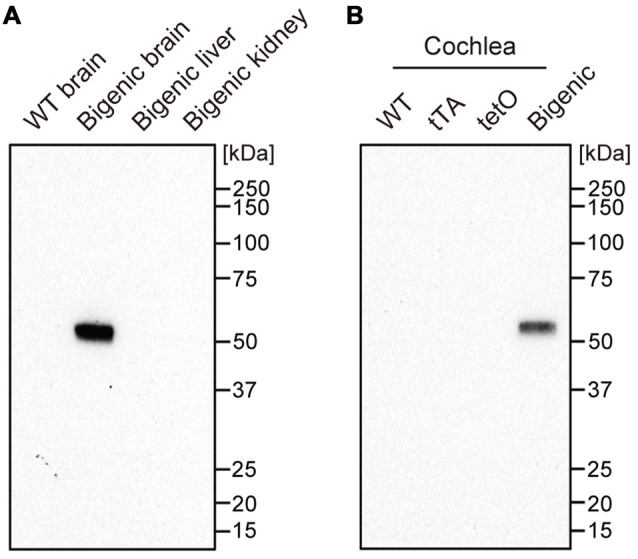
Expression of the channelrhodopsin-2 (ChR2)(C128S) protein in the cochlea. Displayed in **(A,B)** are the results of western blot analysis. A monoclonal anti-green fluorescent protein (anti-GFP) antibody was used to detect ChR2(C128S) fused to enhanced yellow fluorescence protein (EYFP) in several tissues of different mouse lines as described above *each panel*; 4.5 μg of each protein lysate was assayed. WT: wild-type. See Supplementary Figure S1 showing the raw image data of immunoblotting and Ponceau staining of the analyzed polyvinylidene difluoride membrane.

Transgenic mice expressing blue-light-gated ChR2(C128S) were generated by crossing two mouse lines: tetO mice and tTA mice (see “Materials and Methods” Section). The C128S mutant of ChR2 shows much higher sensitivity to (and much less desensitization by) optical stimulation than WT ChR2 does (Berndt et al., 2009). In the bigenic mice, ChR2(C128S), which was tagged with EYFP, is expressed not only in oligodendrocytes of the brain but also in some tissues of the cochlea (Inamura et al., 2012). To confirm the expression of ChR2(C128S) in the cochlea, we carried out western blot analysis with an anti-GFP antibody, which can detect EYFP fused to the channel (Figure 1 and Supplementary Figure S1).

We first tested organs other than the cochlea as control experiments. The antibody did not react with any proteins extracted from a WT mouse brain. On the other hand, a single fragment was detected when the brain of a bigenic mouse at week P10 was examined. The size of the fragment was similar to the expected molecular weight of the EYFP-fused ChR2(C128S) protein (61.7 kDa). No signal was detected in the kidney and liver (both of which contain few neuronal components) of the bigenic mouse (Doerflinger et al., 2003). Second, cochleae in mice of different transgenic lines were analyzed. A single and clear-cut fragment was observed in the lysate of bigenic mice (week P10) as was the case in the brain. Little immunoreactivity was detected in the cochlear samples of WT, tTA and tetO mice.

### Distribution and Cellular Localization of ChR2(C128S) in the Cochlea

**Figure 2:**
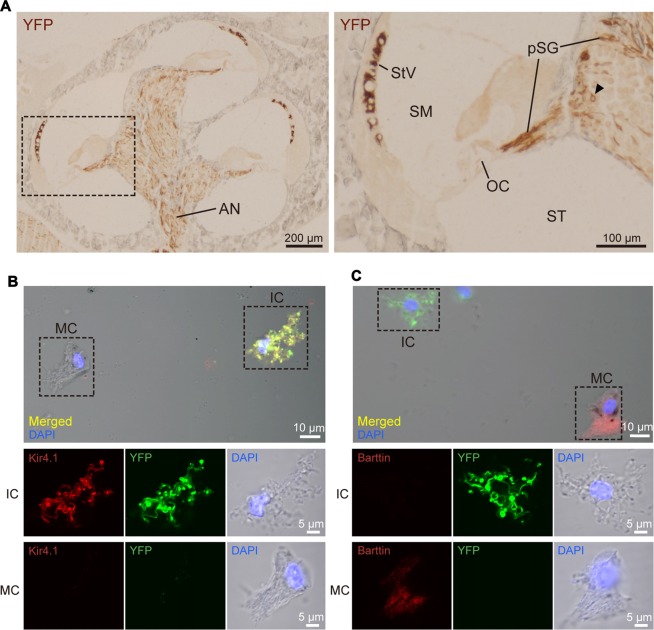
Distribution and cellular localization of ChR2(C128S) in the cochlea. **(A)** Immunohistochemical analysis of a cochlear cross-section from a bigenic mouse. Overall, morphology of the cells and tissues was similar to that in the cochlea of WT mouse, as shown in Supplementary Figures S2, S3. Here, the tissue slices were incubated with an anti-GFP antibody that reacts with EYFP fused to ChR2(C128S). The lateral walls of all turns, modiolus and area of auditory nerve (AN) were stained as depicted in the *left panel*. Higher magnification of the region outlined by the *dashed box* is displayed in the *right panel*. In this image, the positive signal was detected in the stria vascularis (StV), cell body of the spiral ganglion (SG; *arrowhead*), and peripheral and central processes of the pSG. The organ of Corti (OC) did not show staining. Localization of the YFP in the AN, SG and pSG was further precisely determined by confocal microscopic examination (see Supplementary Figure S4). SM, scala media; ST, scala tympani. **(B,C)** Immunolabeling of strial cells. Intermediate cells (IC) and marginal cells (MC) were dissociated from the StV of the bigenic mice and were immunolabeled with antibodies against Kir4.1 **(B)** or barttin **(C)** TRITC (*red*). Expression of ChR2(C128S) was visualized simultaneously with the YFP signal (*green*). Nuclei were stained with DAPI (*blue*). In **(B,C)** areas outlined by *dashed boxes* in the *top panels* are enlarged in *middle* and *bottom panels*. Using a cochlear cross-section, the YFP signal was compared with immunoreactivity of each of Kir4.1, barttin, and glucose transporter 1 (GLUT1), a marker for basal cells (BC), in the StV (see Supplementary Figure S5). Of note, YFP-positive cells in the StV and SG were counted in the assay of Supplementary Figure S6.

The detailed localization of ChR2(C128S) in the cochlea of the bigenic mouse has been elusive (Inamura et al., 2012). We therefore immunohistochemically analyzed cross-sections of the cochlea of the animals at weeks P6–P10 with the anti-GFP antibody. Figure 2A shows representative results of the experiments. YFP immunoreactivity was observed in the lateral wall of all turns of the cochlea and in the modiolus. A positive signal was also detected in the area of the auditory nerve (AN). High-magnification imaging revealed that the immunoreactivity was localized to the StV as well as to the cell bodies and processes of the SG. In the StV, the signal was observed throughout the tissue but was occasionally discontinuous, whereas in the ganglion, the signal was detected in a limited number of cells. No immunoreactivity was observed in the OC.

We next compared the morphology of the cochlea in bigenic mice with that in WT mice by analyzing sectioned tissue (Supplementary Figure S2). Overall, the tissue and cellular structures, including the OC, in bigenic mice appeared to be normal (Supplementary Figures S2A,B). Average thickness of the StV and the number of cell bodies of the ganglion at basal, middle, and apical cochlear turns were not significantly different between the two mouse lines (Supplementary Figure S2C). Furthermore, no abnormalities were observed in the arrangement of the outer and inner hair cells in the OC and in the shape of the hair bundles (Supplementary Figure S3).

The SG and StV are composed of multiple cell types (Raphael and Altschuler, 2003; Hibino et al., 2004). To determine cellular localization of ChR2(C128S) in the cochlea of the bigenic mice, we labeled each cell type with antibodies against marker proteins and compared localization of the signal with that of EYFP by laser confocal microscopy. In the ganglion, ChR2(C128S) was localized to glial cells such as Schwann cells and satellite cells but not to neuronal components (Supplementary Figure S4). This was also the case in the area of the AN (Supplementary Figure S4). The StV consists of marginal, intermediate and basal cells (BC), all of which belong to nonexcitable, nonglial cell types (Raphael and Altschuler, 2003), intermediate cells (IC) are melanocytes (Uehara et al., 2009). Immunolabeling experiments clearly showed that ChR2(C128S) was not expressed in BC (Supplementary Figure S5A). Moreover, YFP was likely to label IC but not MC (Supplementary Figures S5B,C); however, on the cross-sections, these two neighboring cell types were morphologically too difficult to distinguish. We therefore analyzed cells isolated from the StV. YFP fluorescence was strong in the cells labeled with an antibody against Kir4.1, a K^+^ channel exclusively expressed in IC (Ando and Takeuchi, 1999; Hibino et al., 2004), but not detected in the cells labeled with an antibody against barttin, a Cl^−^ channel accessory subunit specifically localized to MC (Estévez et al., 2001; Figures 2B,C). Accordingly, we concluded that ChR2(C128S) is expressed in IC but neither in marginal nor in BC.

On the basis of the above observations, we counted ChR2(C128S)-positive cells in immunolabeled slices of the cochleae from multiple bigenic mice at weeks P6 and P10 (Supplementary Figure S6). Approximately 95% of IC and 5% of satellite cells at each turn expressed the channel.

### Optogenetic Control of Hearing

The functional significance of the activation of ChR2(C128S) in the cochlear StV of bigenic mice has not been analyzed to date. To address this issue, we attempted to measure the ABR using the bigenic mice at weeks P6–10. The animals should undergo the surgical procedure to expose their bulla and be then placed in a supine position for ABR measurements. We confirmed that hearing thresholds of WT mice treated with these processes were comparable to those of the unoperated animals set in a prone position (Supplementary Figure S7).

**Figure 3:**
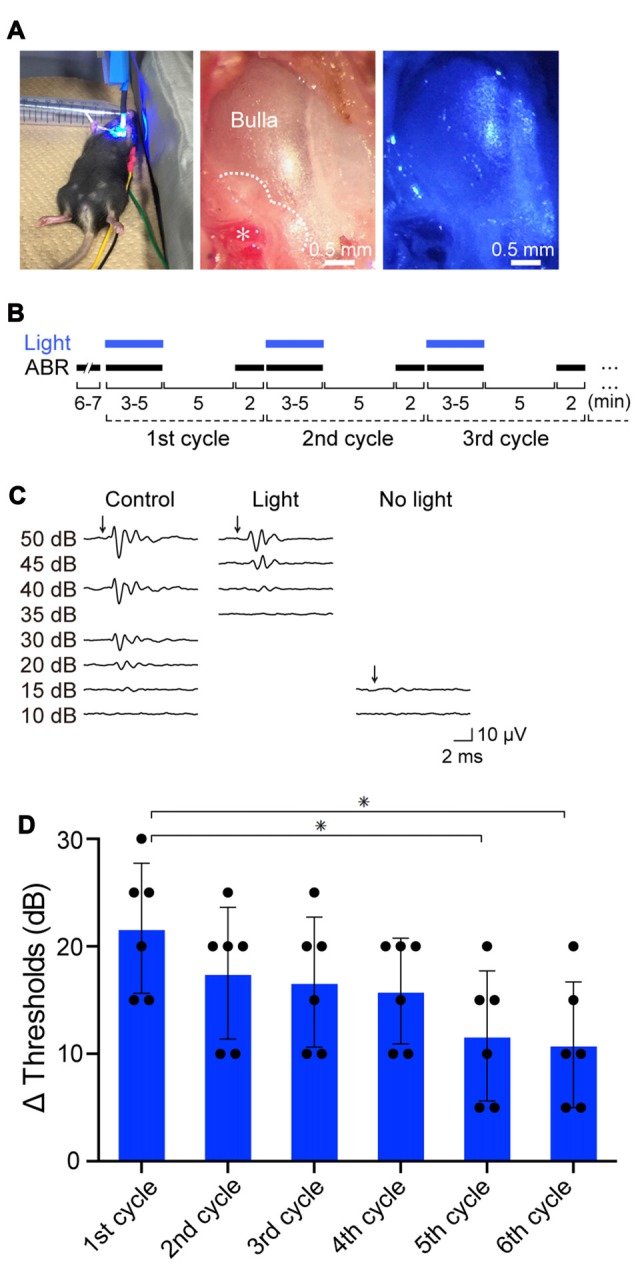
Effects of optical activation of ChR2(C128S) expressed in the cochlea on hearing. **(A)** Illumination of the cochlea. Blue light (463 nm) illuminated the surgically exposed bulla of anesthetized bigenic mice in a supine position (*left panel*). *Middle* and *right panels* show an expanded image of a bulla without and with blue light, respectively. In the* middle panel*, the *asterisk* indicates the stapedial artery running along the cochlear basal turn, and the *dotted line* outlines the cochlea detected through the bulla. As shown in Supplementary Figure S7, hearing thresholds of WT mice that had undergone the surgical process and was set in the supine position resembled those of the intact mice set in a prone position. **(B)** An experimental protocol for examination of the effects of repetitive illumination on hearing. Auditory brainstem response (ABR) measurements and illumination periods are indicated by *black* and *blue bars*, respectively. For details, see “Resuts” and “Materials and Methods” Section. This protocol was determined on the basis of the preliminary experiments shown in Supplementary Figures S8A–C (see *text* as well). **(C)** Representative waveforms of ABR in a bigenic mouse. Click stimuli at the intensities described in the *panel* (≤50 dB sound pressure level (SPL), 5- to 10-dB steps) were provided to the animal under control conditions (*left*), with optical stimulation (*center*) and 5 min after discontinuation of illumination (*right*). **(D)** Effects of repetition of illumination. The cycle of ABR measurements described in **(B)** was performed in bigenic mice sequentially six times; the auditory thresholds elevated by illumination in each cycle are shown (mean ± SD; *n* = 6; *P < 0.01, Dunnett’s test after one-way ANOVA). Dots indicate individual data points. Raw data used for this analysis are shown in Supplementary Figure S8D. Using the similar protocol, auditory thresholds with tone-burst stimuli at six frequencies (4.0, 8.5, 12.5, 16.8, 25.0, and 33.3 kHz) in bigenic mice and other mouse lines were determined (see Supplementary Figure S9).

Initially, we exposed the mice to click sound stimuli in the absence or presence of illumination (Figure 3A). In preliminary experiments, we found that during stimulation of the cochleae with blue light (peak wavelength 463 nm, 0.45 mW/mm^2^) for 3–5 min, the auditory thresholds were elevated from 15-decibel (dB) SPL to 30–40 dB SPL (Supplementary Figure S8A). The thresholds completely recovered 5 min after cessation of illumination (Supplementary Figure S8B). The amplitude of the threshold elevation depended on light intensity, and illumination at 0.45 mW/mm^2^ induced the maximal response (Supplementary Figure S8C). The optical stimulation only minimally influenced the ABR thresholds of WT, tTA and tetO mice (Supplementary Figure S8C). On the basis of these observations, using illumination at 0.45 mW/mm^2^, we precisely evaluated the effects of light-induced activation of ChR2(C128S), including its reversibility and repeatability (Figure 3B). Throughout the experiments, a click stimulus of intensity of ≤50 dB SPL (5- to 10-dB steps, averaging 1000 trials) was used for ABR measurements. All the mice were analyzed for ABR under control conditions, and next, they were subjected to the following series of assays. First, during the ABR measurement (3–5 min), the cochlea was continuously exposed to light. As soon as the auditory threshold was determined, illumination was stopped. After an interval of 5 min, ABR was again measured to assess hearing restoration with stimuli at the intensity that was the same as (or close to) the threshold under control conditions (see “Materials and Methods” Section). The cycle of these experiments was sequentially repeated several times. Figure 3C illustrates representative ABR waveforms obtained from a bigenic mouse by the procedure described above. The auditory threshold under control conditions was 15 dB SPL. Upon first illumination, the threshold increased by 25 dB SPL. Hearing subsequently recovered to the control level after cessation of the illumination. Five subsequent replicates of bigenic mice were tested; the average of the elevated ABR threshold in the first cycle was 21.7 ± 6.1 dB (mean ± SD; *n* = 6). In the second to sixth cycles, light-induced elevation of the auditory threshold and its complete restoration were detected every time, but the degree of elevation gradually decayed (Figure 3D and Supplementary Figure S8D).

We next examined ABR thresholds with tone-burst stimuli at six frequencies (4.0, 8.5, 12.5, 16.8, 25.0 and 33.3 kHz) in the bigenic mice (*n* = 6; Supplementary Figure S9). Throughout the tested frequencies, a reversible light-evoked hearing impairment was detected, as observed in the assays with click stimuli.

Using two bigenic mice, we intended to confirm expression of the ChR2(C128S) protein in the StV and morphological properties of the IC by histological approaches, respectively, after ABR measurements with click sound stimuli under illumination. In these mice, optical stimulation elevated the ABR thresholds by 20 and 25 dB SPL, which fell into the range of the responses observed in the experiments presented in Figure 3D (15–30 dB). As shown in Supplementary Figure S10, immunolabeling of a cochlear cross-section as well as isolated strial cells clearly indicated that ChR2(C128S) was distributed in the StV and coexpressed with Kir4.1 in the IC. Furthermore, the observed histochemical profiles and cellular structures were similar to those revealed in Figure 2 and Supplementary Figures S4A, S5.

### Photoinduced Change in the Endolymphatic Potential

We next intended to determine the source of the reversible repeatable hearing loss induced by illumination in the bigenic mice. First, we focused our analysis on the StV; the membrane potential (*V*_m_) of the IC, which bear ChR2(C128S) in the transgenic mice, plays pivotal roles in generation of a highly positive potential of approximately +100 mV in endolymph, called the EP (Smith et al., 1954; Salt et al., 1987; Takeuchi and Ando, 1998; Takeuchi et al., 2000; Nin et al., 2008, 2016; Lorente-Cánovas et al., 2013). Loss of the EP causes deafness (Marcus et al., 2002; Gow et al., 2004; Rickheit et al., 2008). Before testing a possibility of photoinduced change in the EP, we performed whole-cell patch-clamp recordings of isolated IC to confirm the expression of functional ChR2(C128S).

**Figure 4:**
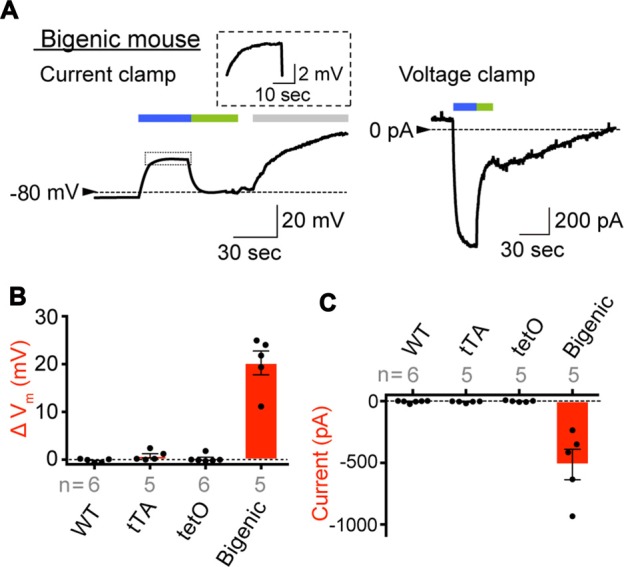
Expression of functional ChR2(C128S) in the IC. **(A)** Representative traces obtained by whole-cell patch-clamp analysis. An IC isolated from the StV of bigenic mice was examined in current-clamp mode (*left*) and voltage-clamp mode (*right*). In the latter condition, the holding potential was −80 mV. The cell was exposed to the lights with peak wavelengths of 463 and 535 nm (0.45 mW/mm^2^) in the periods indicated by *blue* and *green bars* above the traces, respectively. In the current clamp experiment (*left*), an artificial extracellular solution containing Ba^2+^ (3 mM; *gray bar*) was applied to the cell. The trace of the light-evoked current (*boxed region*) is expanded in the *inset*. Current-clamp recording of the intermediated cell optically stimulated for a longer period and that of a MC of a bigenic mouse were also shown in Supplementary Figure S11A. **(B,C)** A summary of photoresponses. IC derived from WT, transcriptional activator (tTA), tetO and bigenic mice were analyzed by the whole-cell patch-clamp method used in **(A)**. Data are shown as mean ± SE for light-evoked changes of membrane potentials **(B)** and maximal amplitudes of inward photocurrent **(C)**. Dots indicate individual data points. In **(B)**, the difference between the resting membrane potential and the steady-state membrane potential during illumination was determined for each cell. Numbers of cells examined are presented under or above each bar. Representative raw traces recorded with the cells from WT, tTA and tetO mice were displayed in Supplementary Figure S11B.

In preliminary experiments, it was difficult to keep the stable seal between the micropipette and cell membrane for more than 10 min, likely owing to fragility of plasma membranes of the cells prepared under our experimental conditions. The opening of ChR2(C128S) evoked by blue light can be immediately aborted by illumination with green light (535 nm; Berndt et al., 2009). We utilized this characteristic to complete the assay within a few minutes. As shown in Figure 4A, an IC dissociated from a bigenic mouse cochlea had V_m_ of −82.9 mV in a current-clamp configuration. This deeply negative potential stems from the dominance of K^+^ conductance involving Kir4.1 on the plasma membrane (Takeuchi and Ando, 1998). We further examined cells obtained from different bigenic mice; the average resting membrane potential (E_res_) was −85.0 ± 1.2 mV (mean ± SE, *n* = 5), which was similar to the E_res_ of cells isolated from the cochleae of WT mice (−83.1 ± 1.1 mV, *n* = 6; *P* = 0.886 as compared to the WT). There was also no statistically significant difference between the E_res_ of the cells from WT mice and that from tTA mice (−85.0 ± 1.7 mV, *n* = 5; *P* = 0.883) or tetO mice (−82.3 ± 2.7 mV, *n* = 6; *P* = 0.992). In Figure 4A, as soon as the blue light illuminated the IC of the bigenic mouse, V_m_ was depolarized rapidly and reached a plateau at −58.8 mV in 20 s. Switching the illumination to green light resulted in recovery of V_m_ nearly to the initial level in 20 s. Finally, application of 3 mM Ba^2+^, a K^+^ channel blocker, caused V_m_ to rise by 39.4 mV, a phenomenon observed in IC but not in other cell types in the StV (Takeuchi and Ando, 1998). Among the five replicate cells assayed, the average of V_m_ elevation in response to the blue light exposure (30 s) was 20.3 ± 2.5 mV (Figure 4B). In one cell, we succeeded in stably recording light-induced depolarization throughout the 3-min blue-light stimulation (Supplementary Figure S11A). The IC were then analyzed in voltage-clamp mode while the membrane potential was held at −80 mV. As shown in Figure 4A, a well-pronounced inward current of 932.4 pA was elicited upon the blue-light illumination and ceased with the green light exposure. Of the five replicate cells examined in current-clamp mode, three cells were successfully analyzed in the voltage-clamp configuration as well. To increase the number of experiments, we additionally examined two cells, which were derived from two different bigenic mice, only in voltage-clamp mode. In summary, the average amplitude of the photocurrent was −513.3 ± 123.2 pA (*n* = 5, Figure 4C). The blue-light-evoked depolarization and inward current were not detected in IC dissociated from WT, tTA, and tetO mice (Figures 4B,C) or in MC dissociated from the bigenic-mouse cochlea (Supplementary Figures S11A,B). In Figures 4B,C note that, among the six IC dissociated from tetO mice, our stable recording in voltage-clamp mode failed in one cell although the other cells as well as all the cells obtained from WT and tTA mice were successfully used in both current- and voltage-clamp experiments.

**Figure 5:**
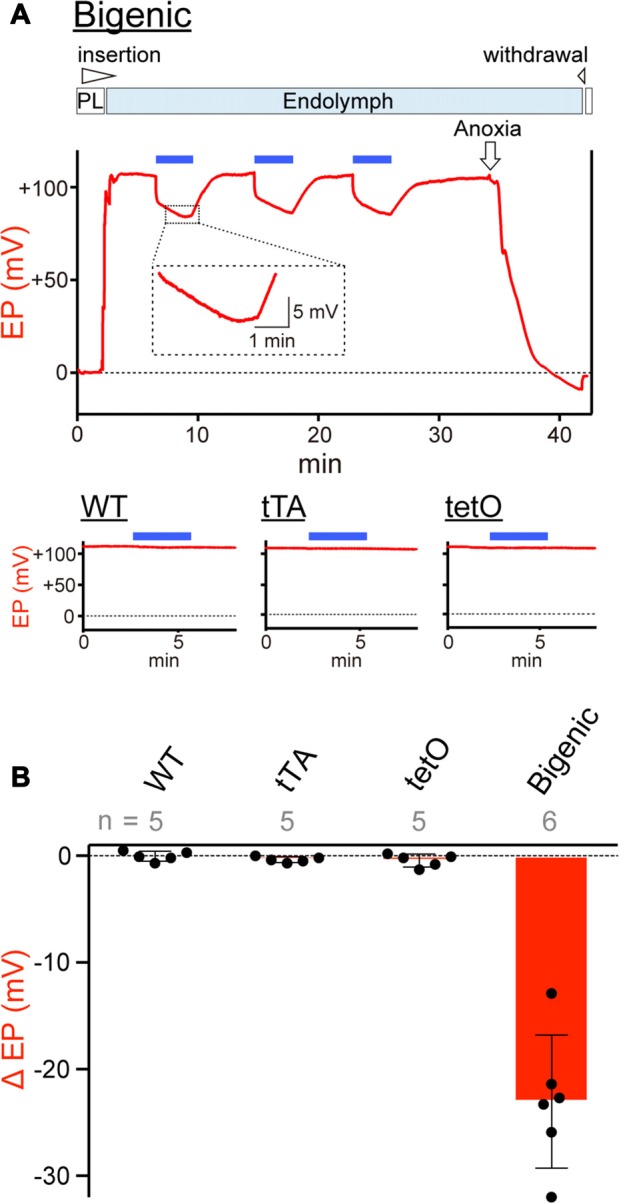
Measurements of the endocochlear potential (EP). **(A)** Representative traces. Top: an example of EP measurement in a bigenic mouse. Here, a glass microelectrode was advanced from perilymph (PL) and held in endolymph. The initial EP was +106.5 mV. While the EP was monitored, the cochlea was stimulated multiple times with light (463 nm) at 0.45 mW/mm^2^ during the period indicated by the *blue bars* above the trace. This light intensity induced a maximal response (see Supplementary Figure S12A). Next, anoxia was applied to the animal and it reduced the EP to −9.1 mV (Kusakari et al., 1978). This observation confirmed insertion of the microelectrode into endolymph. Finally, the electrode was retracted to the perilymphatic space. The region outlined by the *dashed box* is expanded in the *inset*. In this recording, the *wedge* at the top of the *panel* indicates when the electrode was advanced or withdrawn. The *lower three panels* illustrate measurements of the EP in (WT; *left*), tTA (*center*) and tetO (*right*) mice in the presence (*blue bars*) or absence of illumination. Only the recordings in endolymph are shown. Changes of the EP by repetitive and prolonged illumination (six times and 6 min, respectively) were further examined and the results are shown in Supplementary Figures S12B,C. **(B)** Comparison of photoresponses in different mouse lines. Data are shown as mean ± SD for light-induced reductions in the EP. Dots indicate individual data points. Numbers of mice examined are presented above each bar.

On the basis of the results of patch-clamp assays (Figure 4 and Supplementary Figure S11), we tested whether activation of ChR2(C128S) influences the EP *in vivo* (Figure 5). In an experiment shown in Figure 5A, a glass microelectrode inserted into the endolymph of a bigenic mouse revealed a highly positive EP of +106.5 mV under physiological conditions. This value (bigenic mice: +108.2 ± 3.3 mV (mean ± SD), *n* = 6) was similar to the EP value recorded in WT mice (+104.7 ± 4.3 mV, *n* = 5; *P* = 0.7532). This was also the case for the initial EP of tTA (+101.9 ± 5.6 mV, *n* = 5, *P* = 0.8809 compared to the WT) and tetO mice (+102.5 ± 7.2 mV, *n* = 5, *P* = 0.9384 compared to the WT). As indicated in Figure 5A, the cochlea of a bigenic mouse was exposed to blue light for 3 min while the EP was monitored. As soon as the illumination began, the EP abruptly decreased by 15.3 mV with a time constant (τ) of 0.8 s. Then, the potential slowly changed, with *τ* = 220 s, reaching a plateau at +83.3 mV approximately 2.5 min after the onset of the illumination. In response to termination of the optical stimulation, the EP gradually increased and returned to its initial level in ~5 min. When the cochlea was sequentially illuminated with this procedure two more times, a reversible EP response which was likely similar to the response detected during the first stimulation was observed both times. We tested six cochleae from the bigenic mice; the average reduction in the amplitude of the EP by the initial illumination was 23.0 ± 6.2 mV (*n* = 6; Figure 5B). The reduction in EP induced by light depended on the light intensity and showed a maximum at intensity 0.45 mW/mm^2^ (Supplementary Figure S12A). The photoreduction in the EP was barely detectable in the cochleae of WT, tTA and tetO mice (Figures 5A,B). Next, a combination of the optical stimulation (3 min) and its discontinuation (5 min) was applied to the same cochlea sequentially six times (Supplementary Figure S12B). The reversible EP reduction was observed every time, but the change in amplitude gradually declined as the stimuli were repeated. Similar decaying responses were observed in the ABR measurements with repetitive illumination procedures (see Figure 3D). These phenomena are likely to stem from desensitization of ChR2(C128S) to photoactivation. In support of this idea, when hippocampal pyramidal neurons exogenously expressing ChR2(C128S) are stimulated repeatedly, the photocurrent amplitude gradually decreases (Schoenenberger et al., 2009). Moreover, in a bigenic mouse, when the cochlea was continuously illuminated for 6 min, the EP decreased from +108.2 to approximately +75 mV in ~3 min and remained at approximately this potential throughout the rest of the recording (Supplementary Figure S12C).

The EP depends primarily on the membrane potential across the IC (Salt et al., 1987; Takeuchi et al., 2000) although it also involves potentials across the apical and basolateral surfaces of MC and the membranes of fibrocytes (FC) of the spiral ligament (Nin et al., 2008, 2016). The amplitude of the light-induced change of intermediate cells’ membrane potential (20.3 ± 2.5 mV, *n* = 5; Figure 4B) resembled that of the EP (23.0 ± 6.2 mV, *n* = 6, for initial illumination in Figure 5B). This observation suggests that disruption of the electrical properties of IC is responsible for the EP reduction.

Using the other two bigenic mice, we again confirmed expression of the ChR2(C128S) protein in the StV and morphological properties of the IC, respectively, after EP measurements in the presence of illumination. In these cases, the optical stimulation reduced the EP by 31.3 and 36.2 mV in respective animals, as in the experimental results presented in Figure 5B (range of the reduction: 12.9–32.0 mV). As illustrated in immunohistochemical and immunolabeling assays in Supplementary Figure S13, ChR2(C128S) was detected in the StV and was coexpressed with Kir4.1 in the IC. These profiles as well as the cellular structure were similar to the findings in Figure 2 and Supplementary Figures S4A, S5.

### Effects of Optical Stimulation of the Cochlear Glial Cells on Hearing

We finally determined whether activation of ChR2(C128S) expressed in glial cells, which surround neuronal cell bodies and processes of the cochlear SG and AN fiber, contributes to the photoinduced hearing loss in the bigenic mice (for the channel’s localization, see Figure 2A and Supplementary Figure S4). First, in each ear, the interior of the bulla, including the cochlea, was treated with bumetanide (250 μM), a blocker of a Na^+^,K^+^,2Cl^−^-cotransporter (NKCC), for 10 min (see “Materials and Methods” Section). This transporter is expressed in MC and is intimately involved in ion homeostasis of the StV (Kusakari et al., 1978; Wangemann et al., 1995). Subsequently, the EP measured with a glass microelectrode inserted into endolymph registered a value of −7.1 ± 1.7 mV (*n* = 5; mean ± SD; Higashiyama et al., 2003), indicating sufficient dysfunction of the stria. In this setup, activity of hair cells must also be markedly suppressed because it depends on the EP (Honrubia and Ward, 1969). The nervous system in the cochlea was then directly stimulated with currents of varying amplitudes generated by a bipolar electrode while the brainstem response was measured, in an assay called eABR measurement. This method has been validated to exclusively evaluate the function of neuronal components of the cochlea (Yamane et al., 1981).

**Figure 6:**
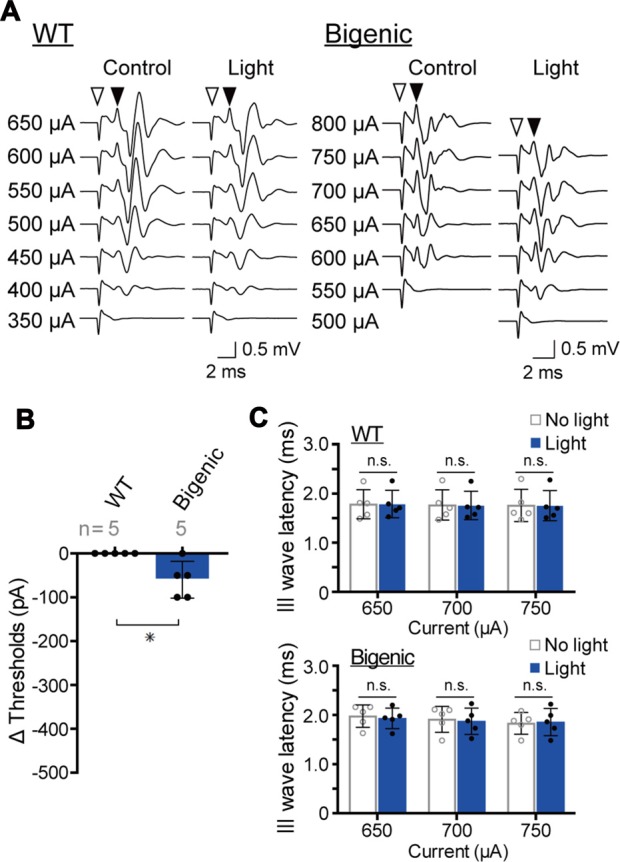
Measurements of the electrical ABR (eABR). **(A)** Representative waveforms of eABR in WT and bigenic mice (*left* and *right*, respectively; average, 200 trials). For eABR measurements, cochleae, which had been treated with bumetanide, were electrically stimulated without (control) or with (light) illumination. Amplitudes of applied currents are shown to the *left* of the traces. *Open arrowheads* indicate the initiation of electrical stimulation. *Filled arrowheads* point to the presumed III wave. **(B)** Changes in eABR thresholds under the influence of optical stimulation. Negative values indicate a decrease in the thresholds in response to illumination. Data are shown as mean ± SD. The number of mice examined is shown above each bar. *P < 0.05 as determined by unpaired two-tailed Student’s *t* test. **(C)** Changes in presumed III wave latency under the influence of optical stimulation. The latency was the interval between the onset of the electrical stimulation and the time point of the peak of the presumed III wave (see **A**). Data are shown as mean ± SD (*n* = 5) with individual values (*dots*). In WT and bigenic mice, values acquired with each current stimulation under control conditions (no light) did not statistically differ from those under illumination (light; n.s.; two-way ANOVA with Bonferroni’s correction).

Among the five WT mice tested, the amplitude of a minimum current inducing a brainstem response varied from 350 to 600 μA (Supplementary Figure S14A). These thresholds were unchanged by blue-light illumination of the cochlea, as illustrated in Figures 6A,B and Supplementary Figure S14A. After that, we examined the five bigenic mice by this method (Figure 6A). The measured eABR threshold ranged from 400 μA to 650 μA (Supplementary Figure S14B). Upon optical stimulation, the thresholds of four mice significantly decreased (by 50 or 100 μA), whereas the threshold of one animal did not alter (average: 60.0 ± 41.8 μA (mean ± SD), *n* = 5) (Figure 6B and Supplementary Figure S14B). These results strongly support the idea that ChR2(C128S) in the cochlear glial cells is functional. We then analyzed the presumable III wave latency representing an interval between the initiation of the electrical stimulation and excitation of the superior olivary nucleus (stimulation currents: 650, 700 and 750 μA). As in the results from WT mice, only a small change in the latency was detected with optical stimulation in the bigenic mice (Figure 6C). Overall, the activity of the nervous system is likely to be slightly enhanced by the illumination of the cochlea, as reported to be the case in the brain (Yamazaki et al., 2014).

ChR2(C128S) was expressed neither in the OC containing hair cells and supporting cells nor in tissues other than the StV and the nervous system (Figure 2A). This observation, as well as the results of the eABR measurements (Figure 6), reinforce the notion that the light-induced reversible hearing loss in bigenic mice stemmed from the EP reduction caused by a change in the membrane potential of the strial IC but not from other elements. In line with these data, the ABR thresholds are elevated by ~1 dB SPL as the EP declines by 1 mV (Schmiedt et al., 2002), and in the present study, optical stimulation of the bigenic-mouse cochleae changed the thresholds by 21.7 ± 6.1 dB (*n* = 6 (mean ± SD); for initial illumination in Figure 3D) and the EP by 23.0 ± 6.2 mV (*n* = 6; for initial illumination in Figure 5B).

## Discussion

Here, we demonstrated that genetic introduction of ChR2(C128S) driven by the PLP promoter results in expression of the functional channels in cochlear IC that are classified into nonexcitable cells (Figure 2). It is again noteworthy that these cells are the melanocytes derived from the neural crest and belong to a nonglial cell type (Raphael and Altschuler, 2003; Uehara et al., 2009; Mayor and Theveneau, 2013). In addition, to our knowledge, animal models of deafness that are based on optogenetics have not yet been generated. Altogether, the present study has expanded the repertoire of optogenetic targets.

**Figure 7:**
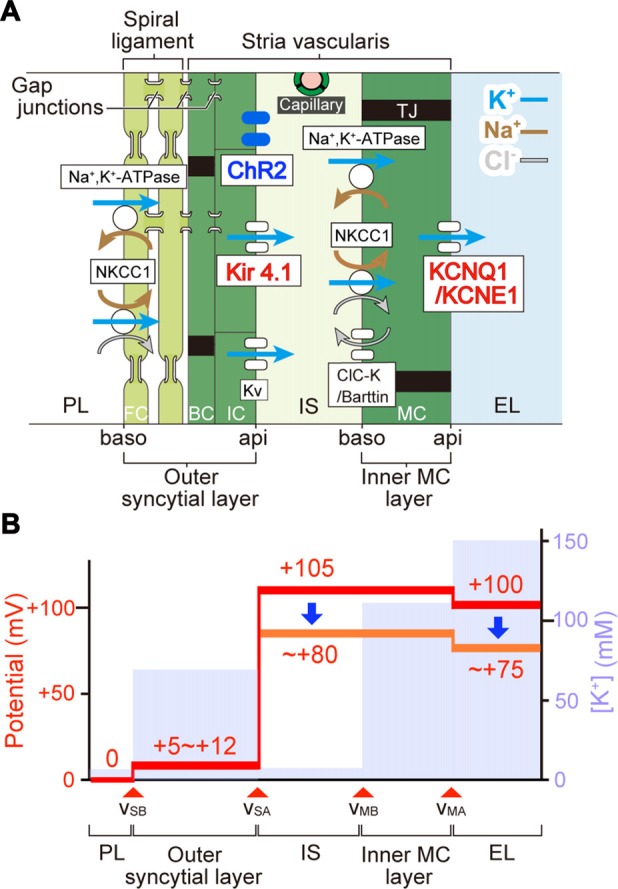
The mechanism underlying photoreduction in the EP. **(A)** Cellular components of the lateral cochlear wall. The lateral wall consists of the StV and spiral ligament. MC in the StV constitute a monolayer. IC and BC in the stria and fibrocytes (FC) in the ligament form an electrochemical syncytium through gap junctions. Therefore, the lateral wall is composed of two functional layers, the inner MC layer and the outer syncytial layer. In this arrangement, the apical (api) and basolateral (baso) surface of the syncytial layer are formed by the membranes of IC and FC, respectively. Between the two layers lies the 15-nm intrastrial space (IS) and capillaries. In bigenic mice, ChR2(C128S) is expressed in the IC membranes (i.e., syncytial apical surface). Ion channels and transporters involved in formation of the EP are shown. Note that the apical surfaces of the syncytial and MC layers are dominated by K^+^ channels Kir4.1 and KCNQ1/KCNE1, respectively. Legend: NKCC1, Na^+^,K^+^,2Cl^−^-cotransporter type 1; K_V_, voltage-dependent K^+^ channel; ClC-K, ClC-Ka/Kb Cl^−^ channel; TJ, tight junction; PL, perilymph; EL, endolymph. **(B)** Electrochemical properties of the lateral wall. Values of the potential (*red*) and [K^+^] (*pale blue*) within the lateral wall are displayed as predicted by our electrophysiological measurements of guinea pig cochleae under physiological conditions (Nin et al., 2008; Yoshida et al., 2016). *v*_SB_, *v*_SA_, *v*_MB_, and *v*_MA_ indicate the membrane potentials across the basolateral and apical surfaces of the syncytial layer and across the basolateral and apical surfaces of the MC layer, respectively. During *in vivo* recordings, the potential of PL is defined as 0 mV. IS potential (ISP) is highly positive. A large difference in potentials across the apical surface of the syncytial layer forms a major fraction of the ISP and corresponds to the IC membrane potential that is deeply hyperpolarized relative to the neighboring extracellular space: IS. Optical stimulation of ChR2(C128S) depolarizes the IC (see Figure 4) and therefore likely reduces the ISP. This change seems to result in the decline of the EP (see Figure 5). Because the values of light-evoked changes of the IC membrane potential are similar to those of the EP (see Figures 4, 5), differences in potentials across the MC layer and the basolateral surface of the syncytial layer are expected to be altered only minimally.

In the StV, intermediate-cells’ membrane is deeply hyperpolarized with reference to the neighboring extracellular solution due to dominance of K^+^ conductance (Salt et al., 1987; Ikeda and Morizono, 1989). This membrane potential is believed to underlie the EP under physiological conditions (Figure 7; Salt et al., 1987; Takeuchi et al., 2000; Nin et al., 2008; Hibino et al., 2010). Kir4.1 is a K^+^ channel abundantly expressed in the IC (Figure 7A) and therefore likely to be deeply involved in formation of the EP (Ando and Takeuchi, 1999; Hibino et al., 2004; Nin et al., 2016). Analyses of Kir4.1 by conventional pharmacological and gene-targeting approaches have suggested that disruption in the electrical homeostasis of IC contributes to deafness. As K^+^ channel blockers such as Ba^2+^, Cs^+^ and quinine are vascularly applied to animals, the EP markedly decreases (Marcus et al., 1985; Takeuchi et al., 1996; Kakigi et al., 2002). Nevertheless, because the blockers are not specific to Kir4.1, they may affect K^+^ channels expressed in other cell types. Kir4.1-null mice, which are deaf, not only show severe loss of the EP but also harbor collapse of the endolymphatic space and degeneration of the OC and SG (Marcus et al., 2002; Rozengurt et al., 2003). This morphological change does not rule out the possibility that factors other than disruption of the membrane potential of IC may also contribute to hearing impairment. On the other hand, the present work indicated that in the StV of bigenic mice, ChR2(C128S) expression was limited to IC (Figures 2B,C and Supplementary Figure S5). Therefore, the membrane potential of these cells was likely to be selectively controlled by optogenetic stimulation. In addition, when the cochleae of bigenic mice were exposed to blue light, the reduction in the EP resembled the change in membrane potential of isolated IC (~ 20 mV for each case; Figures 4B, 5B). Moreover, no morphological abnormality was detected in the cochlea of bigenic mice (Supplementary Figures S2, S3), and the activity of their nervous system was not impaired by illumination (Figure 6). These lines of evidence clearly and directly indicate pathophysiological significance of the IC in deafness. Nonetheless, careful observations revealed that the values of the photoresponse of the EP (23.0 ± 6.2 mV, *n* = 6; for initial illumination in Figure 5B) were slightly different from those of the membrane potential of IC (20.3 ± 2.5 mV, *n* = 5; Figure 4B). This moderate inconsistency may have the following cause. Cohen-Salmon et al. (2007) demonstrated that mice lacking connexin 30 (Cx30), a component of gap junctions in the lateral wall, show a reduced EP owing to disruption of the barrier function of the endothelial cells within the StV (see Figure 7A). The disruption seems to be caused by an increased amount of homocysteine in the stria. Those authors also proposed that the impaired homeostasis of this amino acid may depend upon imbalance of the osmolarity milieu within the stria of the knockout mice. In our bigenic mice, opening of ChR2(C128S) induces influx of cations and thereby it may change the osmolarity balance between extracellular and intracellular spaces in the StV. This condition may result in a phenotype similar to that in Cx30-null mice, possibly accounting for the aforementioned discrepancy. Again, the difference was only small (<3 mV on average) and therefore may not contradict the direct relevance of disruption of the membrane potential in IC to deafness (see above).

Hearing loss in bigenic mice harboring ChR2(C128S) was characterized as follows (Figure 3 and Supplementary Figure S8). First, after illumination, elevation of ABR thresholds developed rapidly and reached a maximal level in a few minutes. Second, the thresholds returned to control levels when illumination was discontinued. Third, this reversible phenotype was likely inducible at any timing and was found to be repeatable. These features may be, at least in part, similar to the symptoms of idiopathic sudden sensorineural hearing loss (SSNHL) in humans. In clinical practice, this disease is most often defined as hearing loss occurring within 72 h, but it can develop within minutes on some occasions (Lazarini and Camargo, 2006; Johnson, 2013). Moreover, 45% to 60% of patients with idiopathic SSNHL experience restoration of their hearing ability even without treatment, and some of the patients have recurrent episodes (Lazarini and Camargo, 2006; Kuo and Young, 2012; Jung et al., 2016). It is noteworthy that hypertension and diabetes seem to be risk and prognostic factors associated with SSNHL (Narozny et al., 2006; Teranishi et al., 2007; Chau et al., 2010). In animals, different levels of severity of these diseases can be caused by controlling the administered dose or treatment duration of angiotensin II and streptozotocin (Gilbert et al., 2011; Brand et al., 2013). In this context, if cochleae of bigenic mice at different hypertensive or hyperglycemic stages were stimulated with blue-light illumination at different intensities and schedules and the ABR thresholds were examined, then a relation between severity of the systemic diseases and that of SSNHL could be discussed. For such experiments, the development of an illumination apparatus implantable into the cochlea of a live mouse would be necessary. Thus, our mouse line may be useful for translational research related to sensorineural hearing loss.

## Author Contributions

MPS, FN, TY, SKomune, KO, HI, HT, KFT and HH designed the experiments; TO, SC, TW, SKanzaki and SSakamoto prepared the experimental devices; MPS, TH, FN, GO, KH, SSawamura, SU, MM and TW carried out the experiments; TH, HT and KFT prepared the transgenic mice; MPS, FN, TY, SKanzaki and HH analyzed the data; and MPS, KD, KFT and HH wrote the article.

## Conflict of Interest Statement

The authors declare that the research was conducted in the absence of any commercial or financial relationships that could be construed as a potential conflict of interest.
