# Supplementary material for: Hearing Loss Controlled by Optogenetic Stimulation of Nonexcitable Nonglial Cells in the Cochlea of the Inner Ear
Source: Front Mol Neurosci. 2017 Sep 21;10:300. doi: 10.3389/fnmol.2017.00300 (PMC5616010; doi:10.3389/fnmol.2017.00300)
Supplement: Supplementary file 1 [file Image_1.pdf]

Supplementary Material

Hearing loss controlled by optogenetic stimulation of nonexcitable nonglial cells in the cochlea of the inner ear

Mitsuo P. Sato, Taiga Higuchi, Fumiaki Nin, Genki Ogata, Seishiro Sawamura, Takamasa Yoshida, Takeru Ota, Karin Hori, Shizuo Komune, Satoru Uetsuka, Samuel Choi, Masatsugu Masuda, Takahisa Watabe, Sho Kanzaki, Kaoru Ogawa, Hidenori Inohara, Shuichi Sakamoto, Hirohide Takebayashi, Katsumi Doi, Kenji F. Tanaka, Hiroshi Hibino*

*** Correspondence: Hiroshi Hibino:** hibinoh@med.niigata-u.ac.jp

Supplementary Figures

Supplementary Figures 1, 2, 3, 4, 5, 6, 7, 8, 9, 10, 11, 12, 13, and 14

REFERENCES for Supplementary Data

Supplementary Figure S1

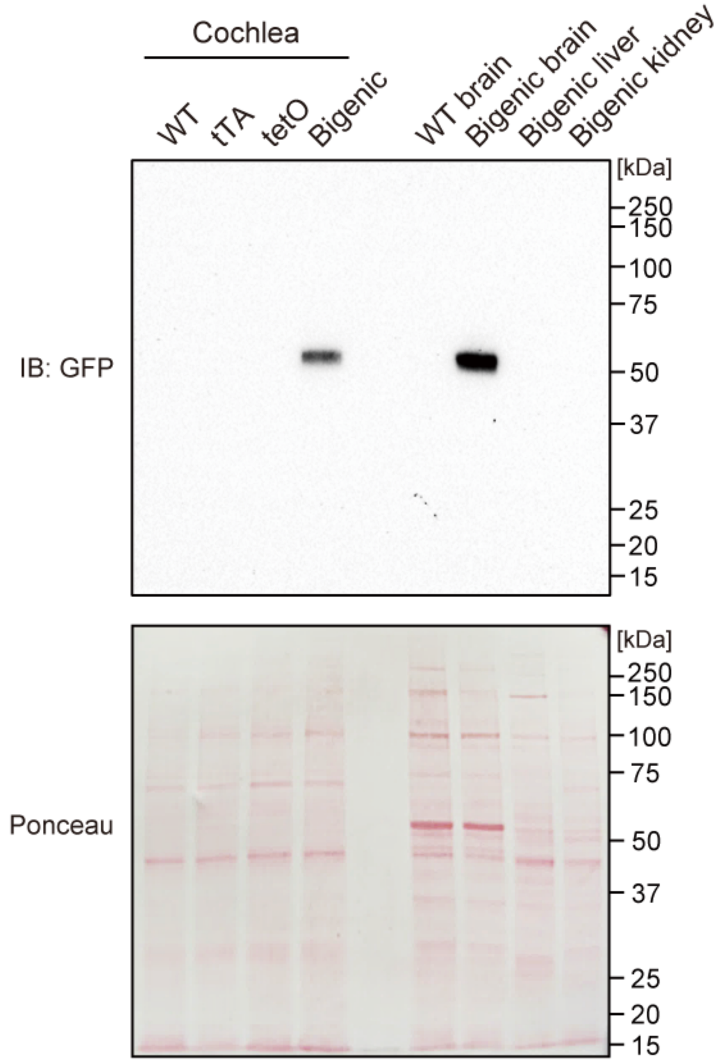

Supplementary Figure S1. The expression profile of the ChR2(C128S) protein in different mouse lines.

The *top panel* shows the raw data of western blot analysis described in **Figure 1**. Protein lysate (4.5 µg) of the cochlea and other tissues in different mouse lines were probed with an anti-GFP monoclonal antibody, which can detect EYFP fused to the ChR2(C128S) protein. The polyvinylidene difluoride membrane used in this analysis was stained in advance with a Ponceau solution as shown in the *bottom panel*. Note that the expected molecular weight of the EYFP-ChR2(C128S) protein is 61.7 kDa (see *top panel*).

Supplementary Figure S2

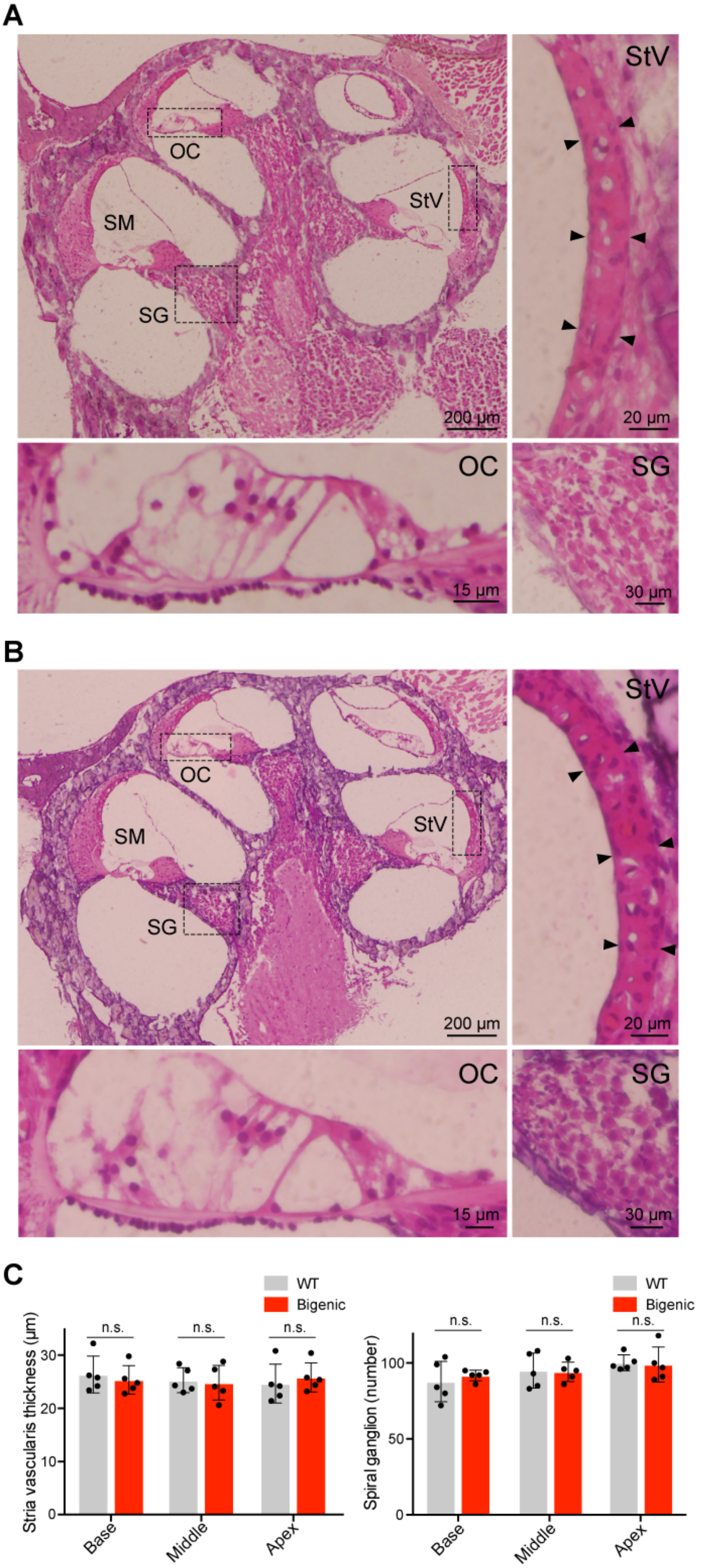

Supplementary Figure S2. Morphology of the cochlea in bigenic mice.

(A and **B)** Cochlear cross-sections of wild-type (WT; ***A***) and bigenic mice (***B***) at week P6. Samples were stained with hematoxylin & eosin and the tissue and cellular structures were examined under a light microscope. The organ of Corti (OC), stria vascularis (StV), and the spiral ganglion (SG) in low-magnification images (*boxed* regions in *left upper panels*) are enlarged in *left lower*, *right upper*, *right lower panels*, respectively. SM, scala media. **(C)** Quantitative analyses. A cross-section of the cochlea obtained from WT and bigenic mice (n = 5; week P6) was examined under the microscope. Average thickness of the StV and number of cell bodies of SG neurons at basal, middle, and apical cochlear turns are shown (mean ± SD). In bar graphs, each set of measurements was plotted. Of note, in each section, "thickness" was defined as an average of three distance measurements perpendicular to the surface of marginal cells facing SM (*arrowheads* in *right upper panels* of ***A*** and ***B***). Statistical analysis involved two-way ANOVA with Bonferroni correction; n.s.: not significant ($P > 0.9999$).

Supplementary Figure S3

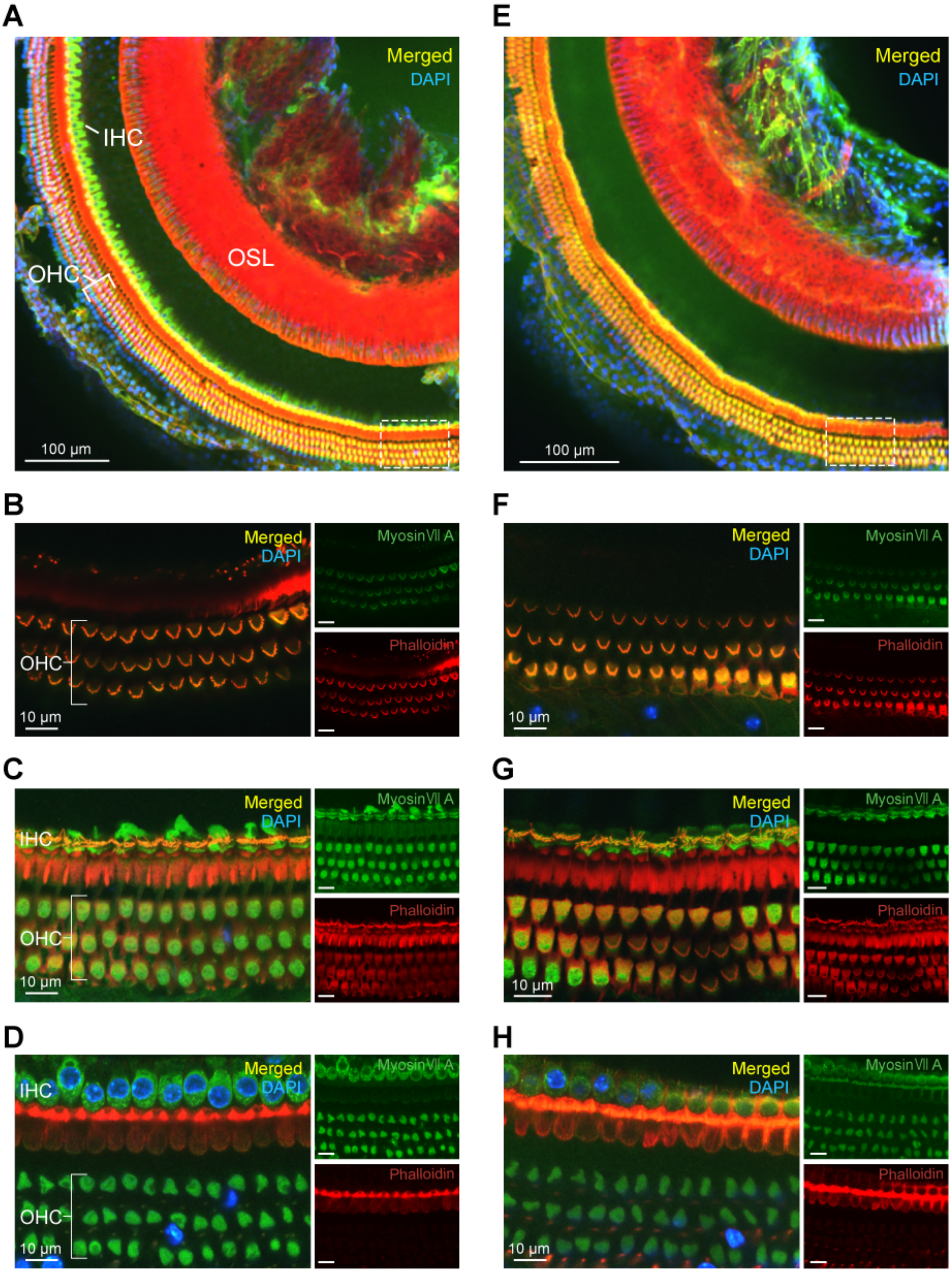

Supplementary Figure S3. Structure of the organ of Corti (OC) in bigenic mice.

A tissue sample of the sensory epithelia, including OC and the spiral limbus at the basal turn, was obtained from cochleae of wild-type (WT) (***A*** – ***D***) and bigenic mice (***E*** – ***H***) and incubated with phalloidin conjugated with rhodamine (*red*) and anti-myosin VIIa antibodies conjugated with Alexa

488 (*green*). Nuclei were stained with DAPI (*blue*). *Panels* ***A*** and ***E*** show low-magnification images of tissue preparations. Regions outlined by *dashed boxes* were further scanned at higher magnification with a confocal microscope focusing on hair bundles of outer hair cells (OHC) (***B*** and ***F***) and inner hair cells (IHC) (***C*** and ***G***) and the somas of these cells (***D*** and ***H***). Note that only a negligible difference in morphology of hair cells and their organelles was observed between the two mouse lines. OSL, osseous spiral lamina.

Supplementary Figure S4

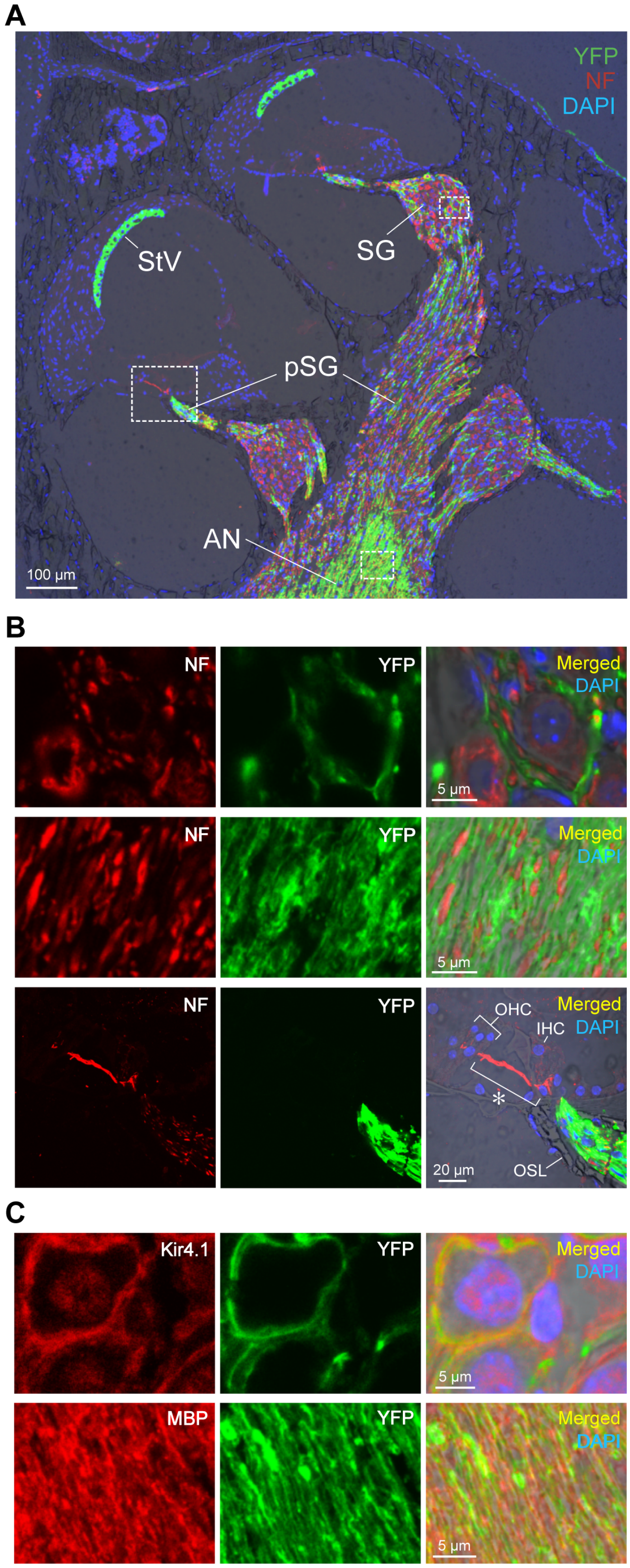

Supplementary Figure S4. Distribution of ChR2(C128S) in the cochlear nervous system.

(A and **B)** Comparison of expression of ChR2(C128S) with that of neurofilament (NF). Cochlear cross-section from a bigenic mouse was reacted with an anti-NF primary antibody and then incubated with a TRITC-conjugated secondary antibody (*red*). ChR2(C128S) was visualized by exciting EYFP fused to this channel protein (*green*). Nuclei were probed with DAPI (*blue*). A low-magnification image shows that ChR2(C128S) is expressed in the spiral ganglion (SG), the processes of spiral ganglion neurons (pSG), and auditory nerves (AN) besides the stria vascularis (StV) (***A***). Regions outlined by *dotted squares* were scanned at higher magnification by means of a confocal microscope and the resulting images are displayed in ***B***. Almost no overlap was detected between NF immunolabeling and YFP signals in cell bodies of SG (*top panels*), AN (*middle panels*), and pSG (*bottom panels*). Of note, a pSG segment that is outside the osseous spiral lamina (OSL) and innervates outer and inner hair cells (OHC and IHC, respectively) is unmyelinated (Nayagam et al., 2011); as depicted in *bottom panels* of ***B***, the processes in this region (*asterisk*) are labeled with an anti-NF antibody, but are free from YFP signals. This observation supports the idea that ChR2(C128S) is expressed in glial cells, but not in neuronal components. **(C)** Comparison of expression of ChR2(C128S) and glial marker proteins in SG and AN. Cross-sections different from the one used in ***A*** and ***B*** were reacted with an antibody against Kir4.1 (*upper panels*), which is specifically expressed in satellite cells (Hibino et al., 1997; Hibino et al., 2004), or an antibody against myelin basic protein (MBP) (*lower panels*), which is localized to Schwann cells and oligodendrocytes. The immune complexes were incubated with a TRITC-conjugated secondary antibody (*red*). ChR2(C128S) was detected with a YFP signal (*green*). Nuclei were stained with DAPI (*blue*). Merged images indicate a significant overlap of the red and green fluorescence, indicating that the channel is expressed in glial cells.

Supplementary Figure S5

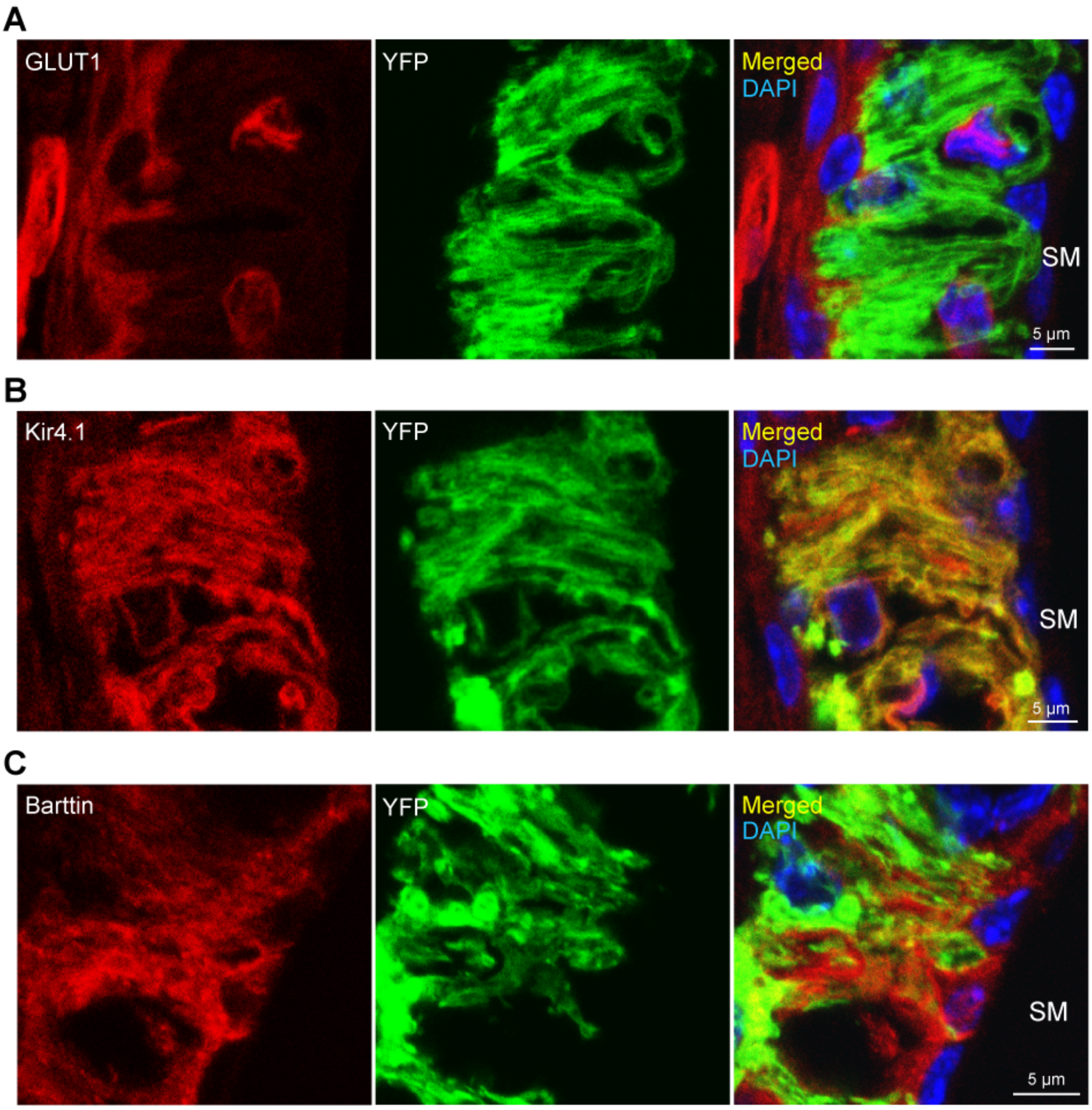

Supplementary Figure S5. Distribution of ChR2(C128S) in the cochlear stria vascularis. *Panels A–C* show confocal microscopic images of the stria vascularis. In the cochlear cross-section samples, GLUT1 in the basal cells (***A***) (Ando et al., 2008), Kir4.1 in the intermediate cells (***B***) (Ando and Takeuchi, 1999; Hibino et al., 2004), and barttin in the marginal cells (***C***) (Estevez et al., 2001), were immunolabeled with specific antibodies (TRITC, *red*) and localization of these marker proteins was compared with that of the YFP signals indicating expression of ChR2(C128S) (*green*). Neither labeling of GLUT1 nor of barttin showed a signal overlapping with the YFP signal. The immunolabeling of Kir4.1showed a "fold"-like appearance and mostly colocalized with YFP labeling. Taken together, these data suggest that ChR2(C128S) is expressed in intermediate cells. Nonetheless, because the membrane of intermediate cells and the basolateral membrane of marginal cells are both

extensively invaginated, and these membranes occupy most of the stria, it was difficult to precisely determine cell type(s) expressing ChR2(C128S) by immunolabeling assays using slices of the cochlea. Nuclei were stained with DAPI (*blue*). SM, scala media.

Supplementary Figure S6

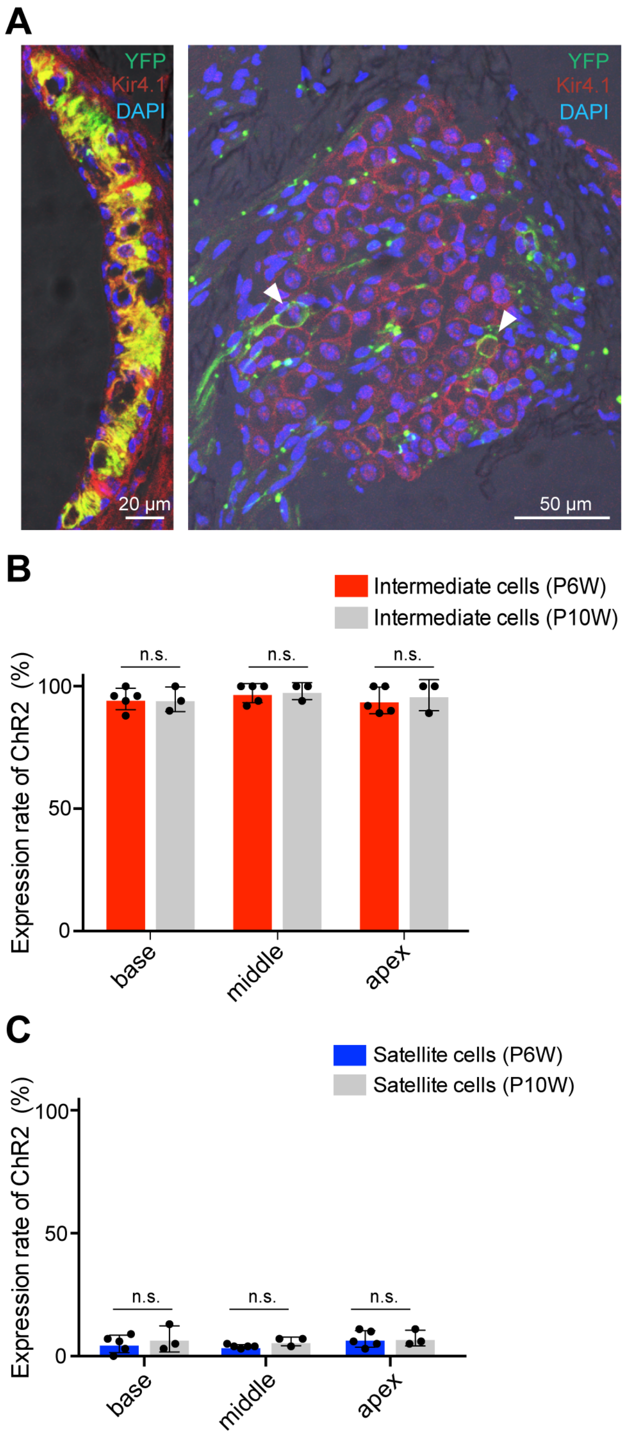

Supplementary Figure S6. Population of ChR2(C128S)-expressing cells in the stria vascularis and spiral ganglion.

(A) An expression pattern of ChR2(C128S). Cochlear cross-sections prepared from bigenic mice were immunolabeled with an antibody against Kir4.1, which is present in intermediate cells of the stria vascularis (*left panel*) and satellite cells of spiral ganglions (*right panel*) (TRITC, *red*). ChR2(128S) was visualized with signals of EYFP fused to this channel protein (*green*). Nuclei were

stained with DAPI (*blue*). The two panels depict merged images (*triple labeling*). In the stria, the majority of intermediate cells express ChR2(C128S) (*left panel*), whereas in the ganglion, a limited number of satellite cells seem to express the channel (*right panel*; *arrowheads*). **(B** and **C)** Quantitative analyses. The total number of intermediate and satellite cells, which were detected by Kir4.1-immunoreactivity, and the number of YFP-positive cells were counted in basal, middle, and apical turns of cochlear samples labeled as shown in ***A***; the respective ratios were obtained and displayed in ***B*** (intermediate cells) and ***C*** (satellite cells) as the mean ± SD with each data point of measurements. For these assays, bigenic mice at week P6 (P6W; n = 5) and week P10 (P10W; n = 3) were used. Statistical analysis involved two-way ANOVA with Bonferroni's correction; n.s.: not significant ($P > 0.9999$).

Supplementary Figure S7

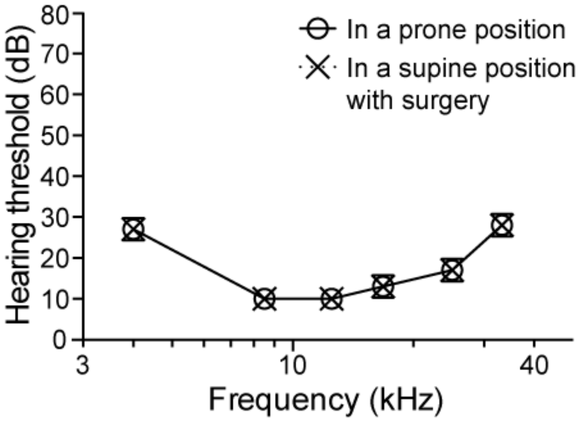

Supplementary Figure S7. ABR measurements in different situations.

Audiograms from wild-type (WT) mice set in a prone or supine position (n = 5 for each group). ABR thresholds were recorded with tone-burst stimuli (4.0, 8.5, 12.5, 16.8, 25.0, or 33.3 kHz). First, the thresholds of the mice were determined with ABR in the prone position. Thereafter, each of these animals underwent a surgical procedure designed to expose the bulla and was subjected to ABR measurement in the supine position. Data are shown as mean ± SD.

Supplementary Figure S8

A

Mouse number	Control ABR	ABR with light
# 1	15 dB	35 dB
# 2	15 dB	40 dB
# 3	15 dB	35 dB
# 4	15 dB	30 dB

B

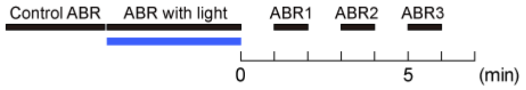

Mouse number	Control ABR	ABR with light	Complete recovery		
			ABR1	ABR2	ABR3
# 5	15 dB	35 dB	○	○	○
# 6	15 dB	35 dB	×	×	○
# 7	15 dB	35 dB	×	○	○
# 8	15 dB	30 dB	×	○	○

C

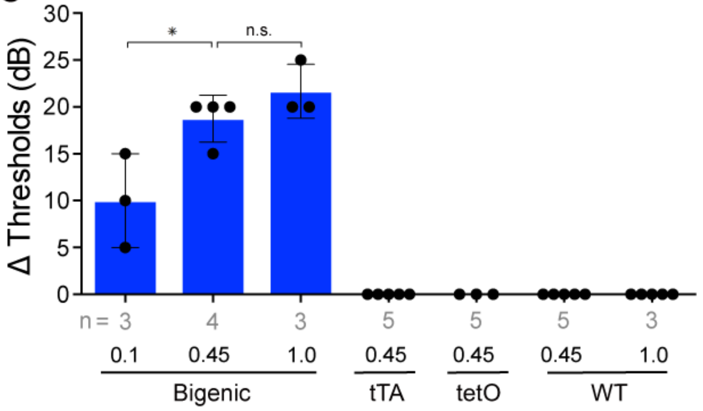

D

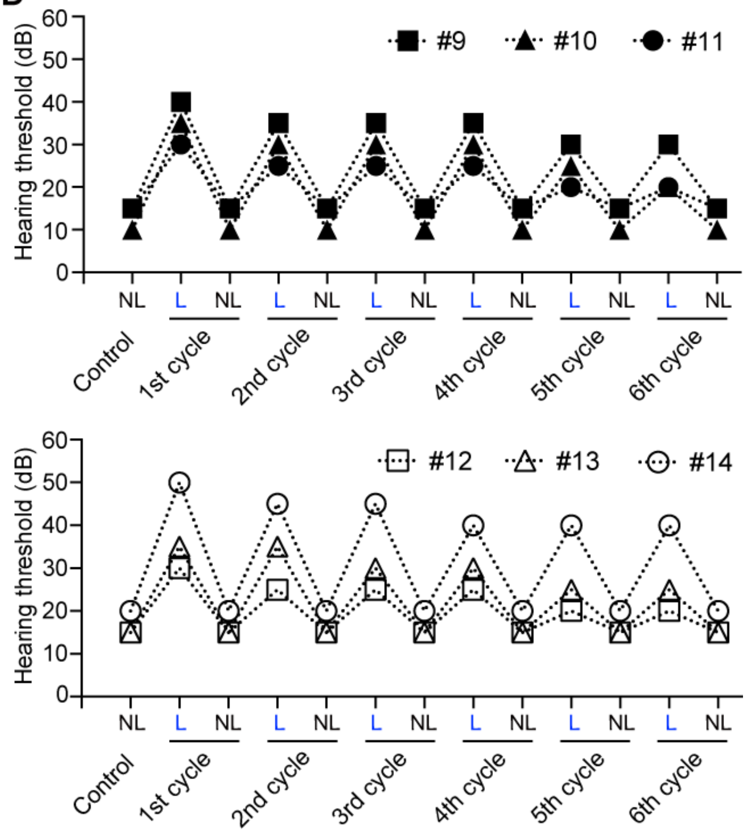

Supplementary Figure S8. Characterization of light-induced elevation of ABR thresholds.

(A) Effects of activation of ChR2(C128S) expressed in the cochlea of bigenic mice. Auditory thresholds of individual mice tested (n = 4) under the conditions described below are presented in the *panel*. For each animal, initially, ABR measurement was performed under control conditions, i.e. without optical stimulation. Thereafter, blue light (peak wavelength 463 nm, 0.45 mW/mm^2) illuminated the cochlea while ABR was recorded. This recording (with illumination) took 3–5 min. Click stimuli were used for all the ABR measurements. **(B)** Restoration of hearing. The experimental protocol is illustrated at the *top*. Four bigenic mice were examined with ABR initially under control conditions and sequentially with optical stimulation of the cochleae (*blue bar*; 3–5 min) as described in the caption of ***A***. Thereafter, illumination was ceased, and ABR measurements using click stimuli at the same intensity as the control auditory threshold were performed three times every 2 min to determine when hearing completely recovered. Each ABR measurement took approximately 1 min (*black bars*). The results on all the mice are displayed in the *Table*. **(C)** Relation between illumination intensity and elevation of auditory thresholds. Displayed are changes in ABR thresholds (mean ± SD and each data point of measurements) when cochleae were exposed to blue light at different intensities (0.1–1.0 mW/mm^2; see under the bars). In this series of assays, bigenic, tTA, tetO, and wild-type (WT) mice were analyzed. The number of the tested animals is shown under the bars. The experimental procedure was the same as the one used in **Figure 3** (also see the ***main text***), except that each cochlea was illuminated only once. *$P < 0.05$ as determined by one-way ANOVA with Tukey's *post hoc* test; n.s.: not significant ($P = 0.5476$). **(D)** Changes of ABR thresholds in repeatedly illuminated bigenic mice. Six mice were tested (mouse #9 to #14). The cycle of ABR measurements described in **Figure 3B** (see the ***main text***) was performed in the mice sequentially six times and the obtained auditory thresholds without or with illumination [NL (no light) and L (light), respectively] in each cycle are shown. Note that, before this series of assays, ABR of individual animals was examined without illumination (control). These data were subjected to the analysis shown in **Figure 3D** in the ***main text***.

Supplementary Figure S9

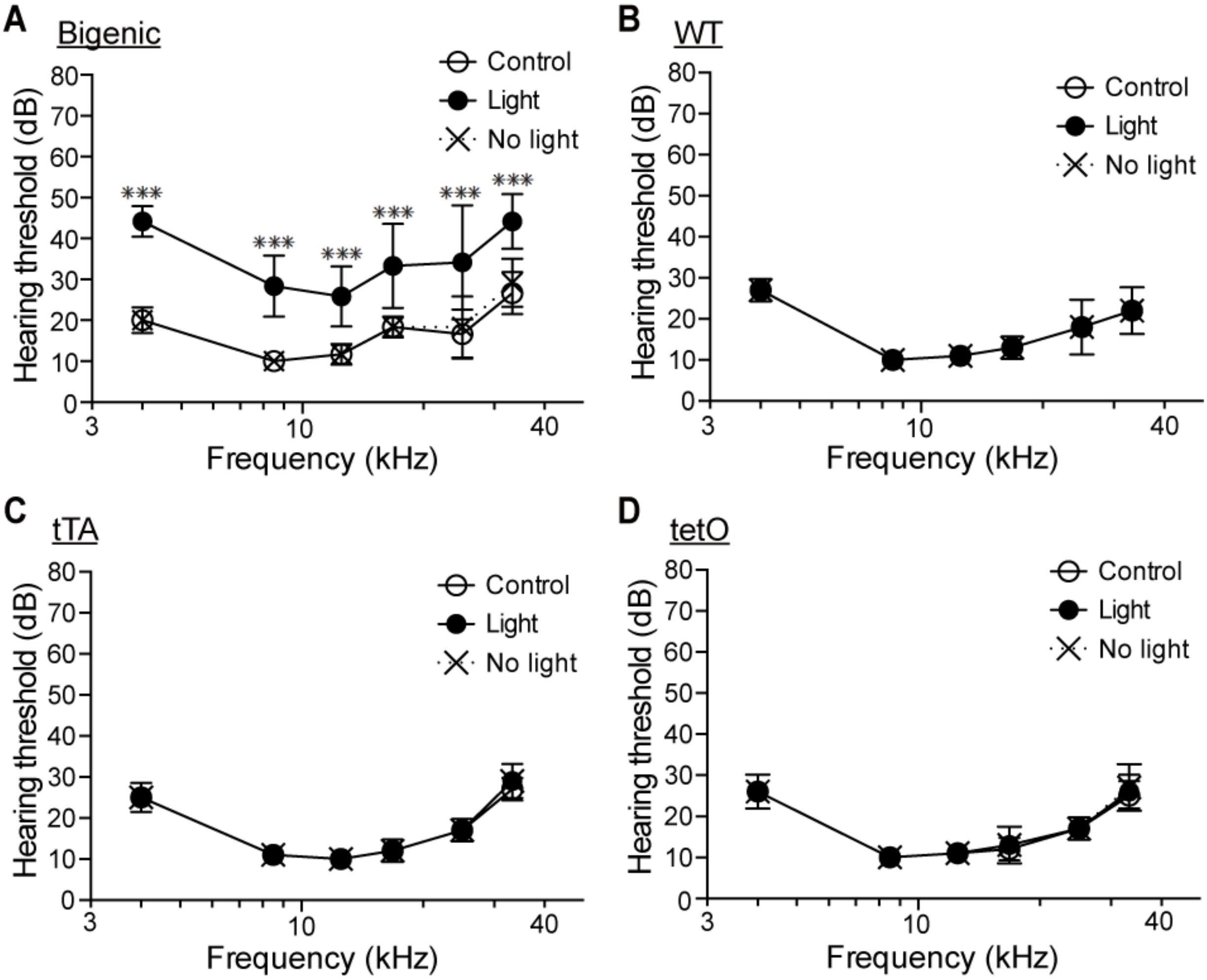

Supplementary Figure S9. The effect of illumination of cochleae on hearing in different mouse lines.

Audiograms of bigenic (***A***; n = 6), WT (***B***; n = 5), tTA (***C***; n = 5), and tetO (***D***; n = 5) mice are shown under control conditions, with illumination of cochleae, and during discontinuation of illumination. Tone-burst stimuli at 4.0, 8.5, 12.5, 16.8, 25.0, and 33.3 kHz were used for determination of ABR thresholds. The protocol was similar to that shown in **Figure 3B**; after determination of the thresholds under control conditions, a cycle of illumination (3–5 min) and its discontinuation (~7 min) with ABR measurements at each frequency was applied to the animals. Data are expressed as mean ± SD. In bigenic mice (***A***), a reversible light-evoked hearing impairment was detected throughout the tested frequencies. During the testing of all the mice, the stimuli began at 4.0 kHz, and the frequency was increased stepwise to 33.3 kHz. Given that elevation of the auditory thresholds determined by means of click stimuli decayed as illumination was repeated (see **Figure 3D**), the effects of illumination at the higher frequencies might have been underestimated in these experiments. Only a small threshold change was induced by optical stimulation at all the tested frequencies in the wild-type (WT), tTA mice, and tetO mice (***B***, ***C***, and ***D***, respectively). ****P*

<0.0001 as determined by two-way ANOVA with Bonferroni's correction.

Supplementary Figure S10

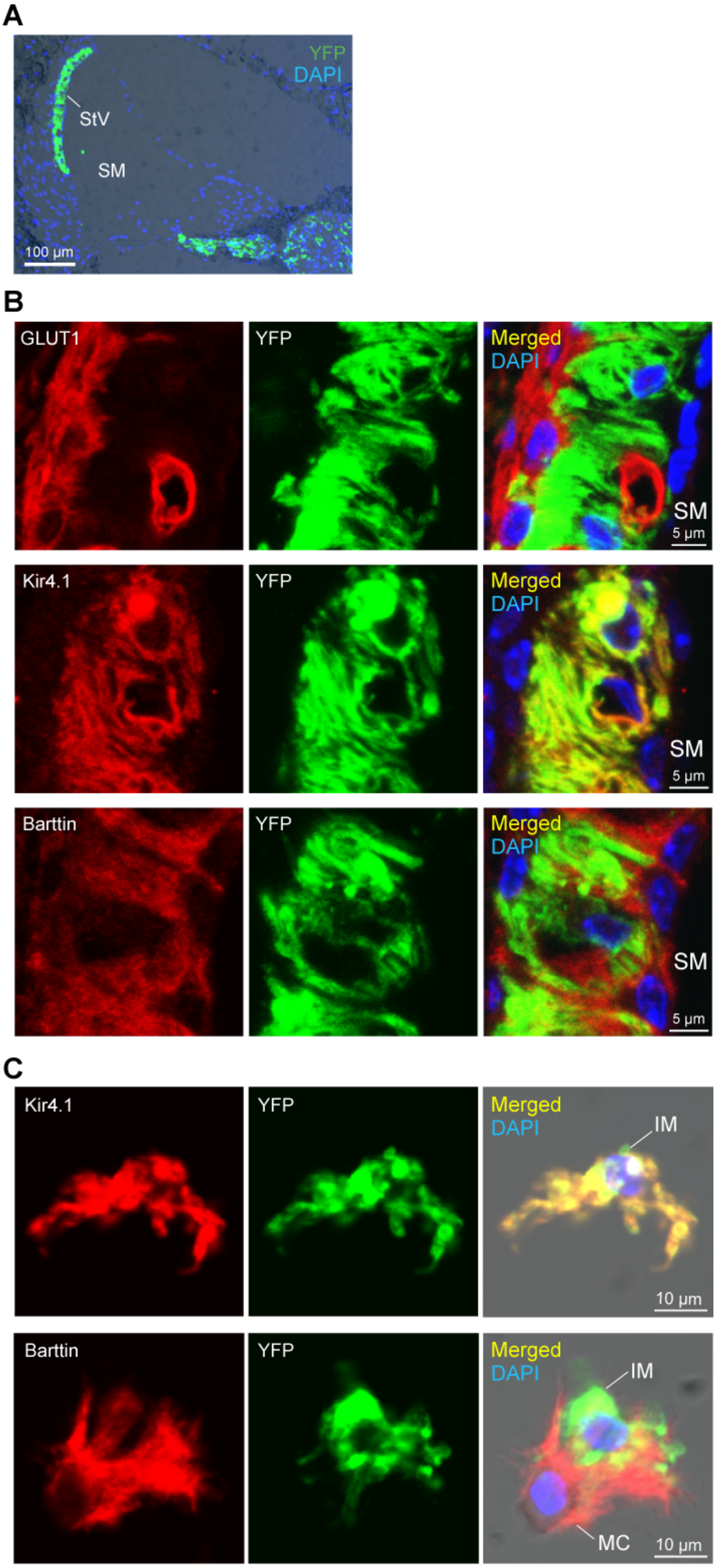

Supplementary Figure S10. The expression profile of ChR2(C128S) after ABR measurements.

ABR thresholds of two bigenic mice were examined in the absence or presence of illumination and the cochlea or cells of the stria vascularis in respective animals were subjected to immunolabeling assays with confocal microscopy. The results of these assays are shown in the *panels*. (**A** and **B**) Cochlear cross-sections obtained from one bigenic mouse. Photoinduced ABR threshold shift of the mouse in question was 20 dB SPL. Panel ***A*** is a low-magnification image of the cochlea. The fluorescence of YFP fused to ChR2(C128S) is visualized as *green color*. StV: stria vascularis, SM: scala media. Panel ***B*** illustrates enlarged images of the stria vascularis. Here, the samples were probed with antibodies against glucose transporter 1 (GLUT1) (*top panels*), Kir4.1 (*middle panels*), and barttin (*bottom panels*) (TRITC, *red*), and localization of these marker proteins was compared with that of the YFP signals (*green*). In ***A*** and ***B***, nuclei were stained with DAPI (*blue*). (**C**) Strial cells isolated from the other bigenic mouse. A photoinduced ABR threshold shift in this mouse was 25 dB SPL. Intermediate cells (IC) and marginal cells (MC) were immunolabeled with antibodies against Kir4.1 (*upper panels*) or barttin (*lower panels*) (TRITC, *red*). Expression of ChR2(C128S) was visualized simultaneously with the YFP signal (*green*). Nuclei were stained with DAPI (*blue*). In the *lower panels*, little YFP fluorescence was detected in a marginal cell.

All these results as well as the tissue and cellular morphology observed in ***A***–***C*** are similar to those in **Figure 2** and **Supplementary Figures S4A** and **S5**.

Supplementary Figure S11

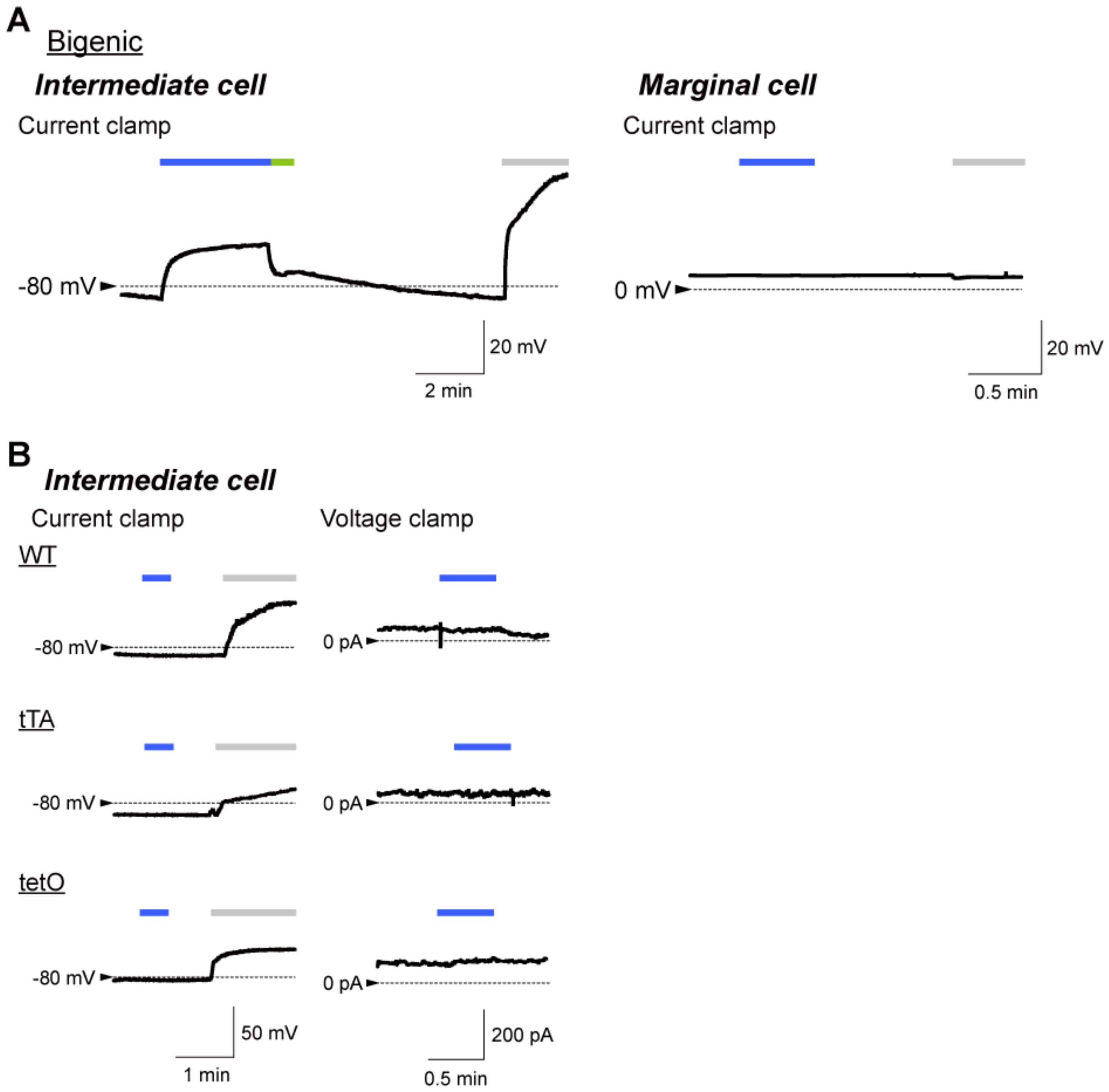

Supplementary Figure S11. Additional whole-cell patch clamp recordings.

(A) Profiles of strial cells in bigenic mice. An intermediate cell (*left panel*) and a marginal cell (*right panel*) were analyzed in current-clamp mode. The cells were stimulated with blue light (463 nm, 0.45 mW/mm^2) when indicated above the traces (*blue bars*). In the assay of the intermediate cell (*left panel*), the illumination was switched to green light (535 nm, 0.45 mW/mm^2; *green bar*) as indicated by the changing color of the *bar*. A K^+ channel blocker, Ba^{2+} (3 mM), was applied to the bath solution (*gray bars* in *both panels*). Note that the duration of blue light stimulation for the intermediate cell was 3 min, which was longer than the period shown in **Figure 4A** (30 s). **(B)** Profiles of intermediate cells prepared from other mouse lines. Cells isolated from wild-type (WT), tTA, and tetO mice were analyzed in current- and voltage-clamp configurations (*left* and *right panels*). Blue light illumination was provided for the cells during periods marked by *blue bars* above

the traces. In the experiments in current-clamp mode, Ba^{2+} (3 mM) was applied to the cells (*gray bars*).

Supplementary Figure S12

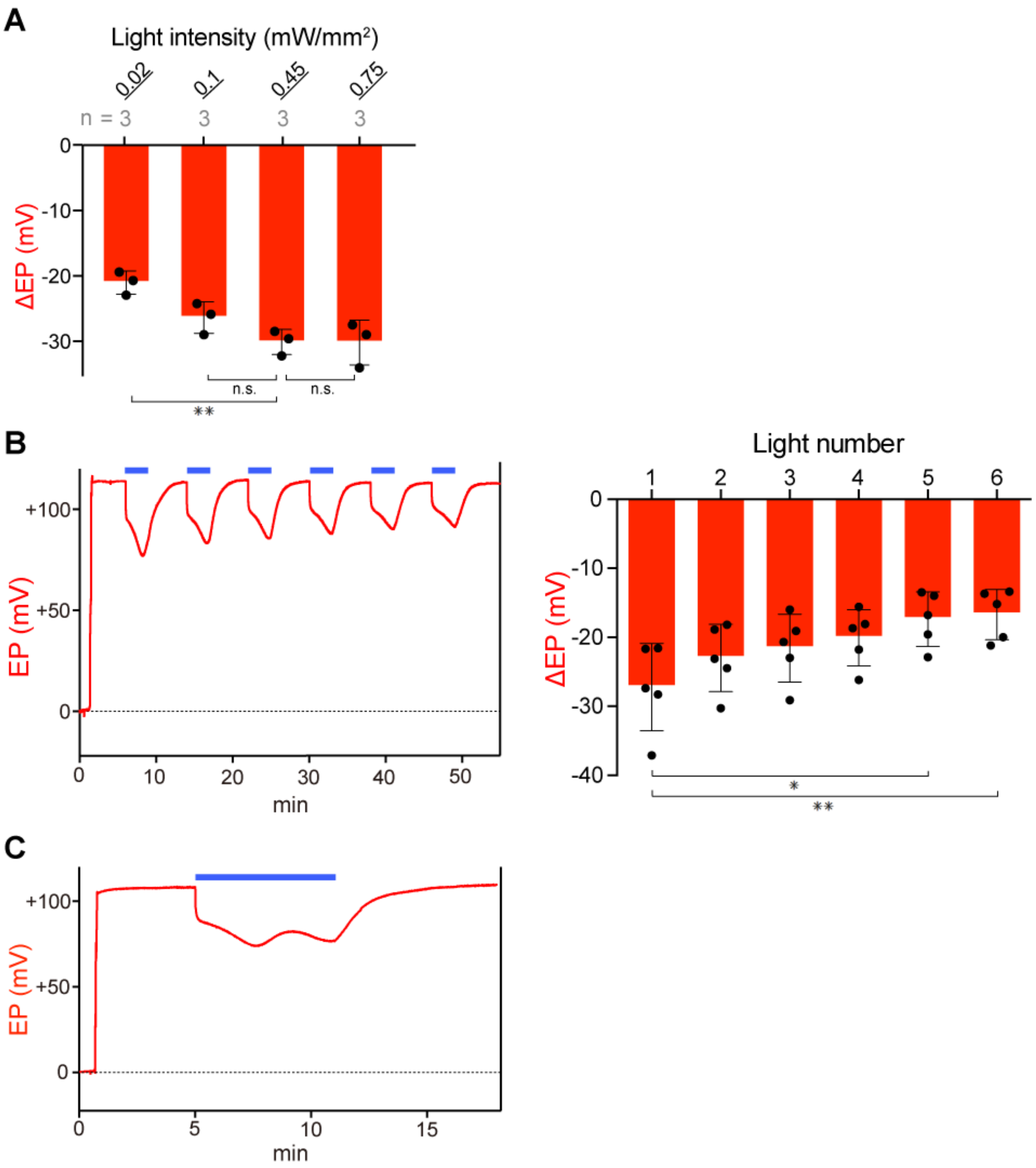

Supplementary Figure S12. Characterization of photoreduction of the endocochlear potential (EP).

(A) Relation between illumination intensity and an EP reduction. Cochleae of bigenic mice were exposed to blue light (463 nm) at different intensities (0.02, 0.1, 0.45, or 0.75 mW/mm^2) for 3 min while the EP was measured by means of a glass microelectrode placed in endolymph. The difference between the initial value of the EP and its steady-state value during illumination was determined, and average values of the evoked EP reduction are shown in the *panel* (mean ± SD and each data point of

measurements). The number of cochleae used for each series of the experiments is presented above the bars; each cochlea was derived from an individual animal and optically stimulated once. The results indicate that the responses were saturated with illumination at 0.45 mW/mm^2. $^{**}P < 0.01$ as determined by one-way ANOVA with Tukey's test; n.s.: not significant (0.1 versus 0.45 mW/mm^2: $P = 0.3199$, 0.45 versus 0.75 mW/mm^2: $P > 0.9999$). **(B)** Effects of repetition of illumination. In one sample depicted in *left panel*, the cochlea of a bigenic mouse was optically stimulated with blue light (463 nm, 0.45 mW/mm^2) six times, as indicated by the *blue bars* above the trace, while the EP was monitored. The interval between stimulations was 5 min. The difference between the EP value immediately prior to the onset of illumination and the steady-state value during illumination was obtained every time in each mouse. Five cochleae from five individual animals were examined by the experimental protocol used in *left panel*, and average values of the potential difference are shown in *right panel* (mean ± SD and each data point). $^{*}P< 0.05$, $^{**}P < 0.01$ as determined by two-way ANOVA with Dunnett's test. **(C)** Effects of prolonged illumination. The cochlea from a bigenic mouse was stimulated with blue light (463 nm) for 6 min during the period indicated by *blue bars* above the trace. The response was saturated at ~3 min after the onset of illumination and thereafter stabilized relatively.

Supplementary Figure S13

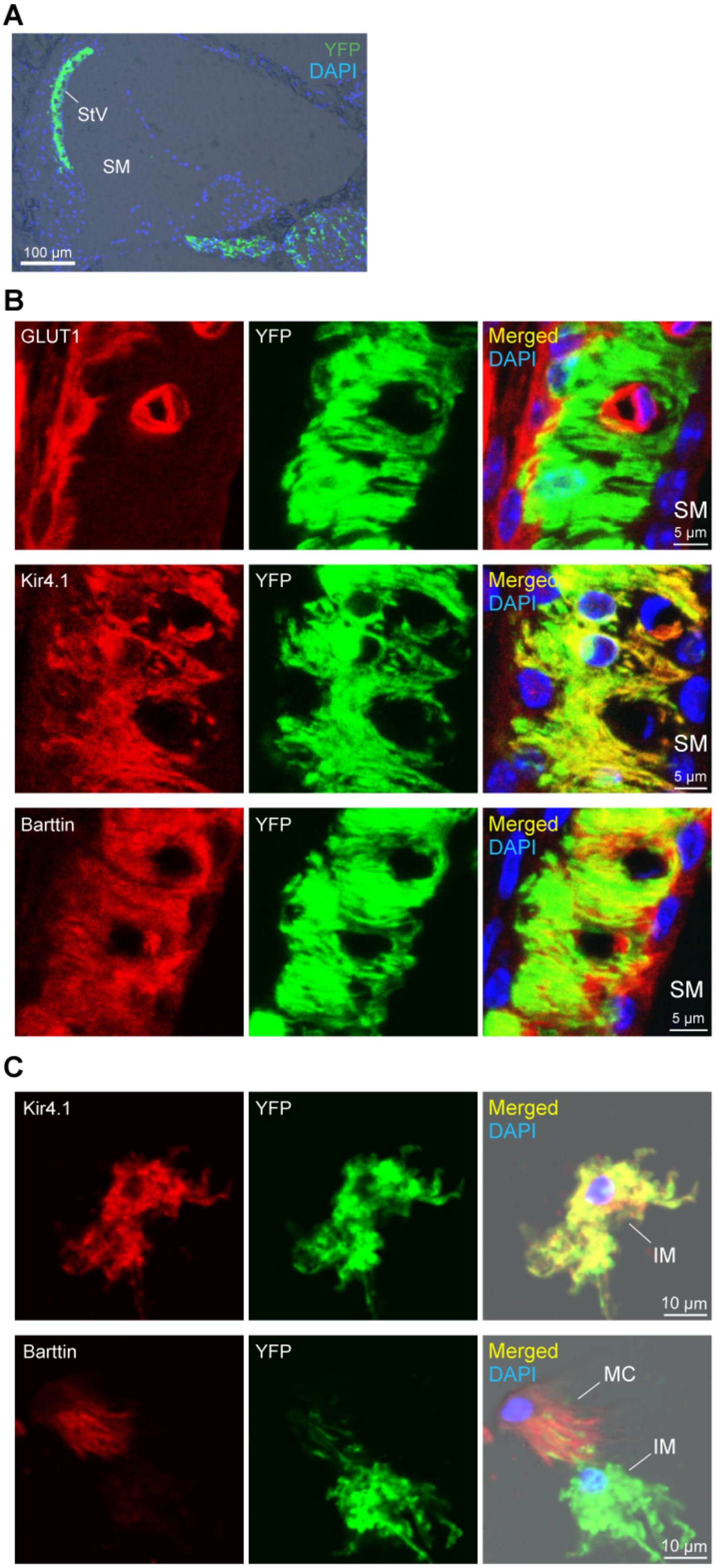

Supplementary Figure S13. The expression profile of ChR2(C128S) after EP measurements. The endocochlear potential in two bigenic mice was measured in the absence and presence of optical stimulation and the cochlea or cells of the stria vascularis in respective animals were subjected to immunolabeling assays. The results are shown in the *panels*. (**A** and **B**) Cochlear cross-sections obtained from one bigenic mouse. A photoreduced EP value of the mouse under study was 31.3 mV. ***A*** is a low-magnification image of the cochlea. Distribution and localization of ChR2(C128S) was visualized with the fluorescent of YFP fused to this channel protein (*green*). ***B*** shows enlarged images of the stria vascularis. Here, the samples were probed with antibodies against glucose transporter 1 (GLUT1) (*top panels*), Kir4.1 (*middle panels*), and barttin (*bottom panels*) (TRITC, *red*), and localization of these marker proteins was compared with that of the YFP signals (*green*). Nuclei were stained with DAPI (*blue*). StV: stria vascularis, SM: scala media. (**C**) Strial cells from the other bigenic mouse. A decrease of the EP by illumination in this mouse was 36.2 mV. Intermediate cells (IC) and marginal cells (MC) were immunolabeled with antibodies against Kir4.1 (*upper panels*) or barttin (*lower panels*) (TRITC, *red*). Expression of ChR2(C128S) was visualized simultaneously with the YFP signal (*green*). Nuclei were stained with DAPI (*blue*). In the *lower panels*, YFP fluorescence was barely detectable in the marginal cell.

All these results as well as the tissue and cellular morphology observed in ***A*–*C*** are similar to those in **Figure 2** and **Supplementary Figures S4A** and **S5**.

Supplementary Figure S14

A

Wild

Mouse number	eABR threshold (μA)	
	Control	with light
# 15	400	400
# 16	500	500
# 17	400	400
# 18	600	600
# 19	350	350

B

Bigenic

Mouse number	eABR threshold (μA)	
	Control	with light
# 20	650	550
# 21	600	550
# 22	600	500
# 23	600	600
# 24	400	350

Supplementary Figure S14. Individual data from the electrical ABR (eABR) analysis.
Initial eABR thresholds without illumination (control) and the thresholds during optical stimulation (with light) were determined in five wild-type (WT) mice (mouse #15 to #19) and five bigenic mice (mouse #20 to #24); individual measurements are shown in ***A*** and ***B***, respectively. These data were used for the analysis in **Figure 6B**.

REFERENCES for Supplementary Data

Ando, M., Edamatsu, M., Fukuizumi, S., and Takeuchi, S. (2008). Cellular localization of facilitated glucose transporter 1 (GLUT-1) in the cochlear stria vascularis: its possible contribution to the transcellular glucose pathway. *Cell Tissue Res* 331(3), 763-769. doi: 10.1007/s00441-007-0495-2.

Ando, M., and Takeuchi, S. (1999). Immunological identification of an inward rectifier K+ channel (Kir4.1) in the intermediate cell (melanocyte) of the cochlear stria vascularis of gerbils and rats. *Cell Tissue Res* 298(1), 179-183.

Estevez, R., Boettger, T., Stein, V., Birkenhager, R., Otto, E., Hildebrandt, F., et al. (2001). Barttin is a Cl- channel beta-subunit crucial for renal Cl- reabsorption and inner ear K+ secretion. *Nature* 414(6863), 558-561. doi: 10.1038/35107099.

Hibino, H., Higashi-Shingai, K., Fujita, A., Iwai, K., Ishii, M., and Kurachi, Y. (2004). Expression of an inwardly rectifying K+ channel, Kir5.1, in specific types of fibrocytes in the cochlear lateral wall suggests its functional importance in the establishment of endocochlear potential. *Eur J Neurosci* 19(1), 76-84.

Hibino, H., Horio, Y., Inanobe, A., Doi, K., Ito, M., Yamada, M., et al. (1997). An ATP-dependent inwardly rectifying potassium channel, KAB-2 (Kir4. 1), in cochlear stria vascularis of inner ear: its specific subcellular localization and correlation with the formation of endocochlear potential. *J Neurosci* 17(12), 4711-4721.

Nayagam, B.A., Muniak, M.A., and Ryugo, D.K. (2011). The spiral ganglion: connecting the peripheral and central auditory systems. *Hear Res* 278(1-2), 2-20. doi: 10.1016/j.heares.2011.04.003.
